# PARP7 inhibition stabilizes STAT1/STAT2 and relieves experimental autoimmune encephalomyelitis in mice

**DOI:** 10.1016/j.celrep.2025.116130

**Published:** 2025-08-12

**Authors:** Jiashu Xu, Tao Yu, Zongwei Yue, Xuan Lu, Yandong Zhang, Lei Wang, Samaneh Shabani Åhrling, Michael R. Smith, Yan Chun Li, Jason Matthews, Hening Lin

**Affiliations:** 1 Department of Chemistry and Chemical Biology, Cornell University, Ithaca, NY 14853, USA; 2 Department of Medicine and Department of Chemistry, The University of Chicago, Chicago, IL 60637, USA; 3 Department of Medicine, The University of Chicago, Chicago, IL 60637, USA; 4 Department of Nutrition, Institute of Basic Medical Sciences, Faculty of Medicine, University of Oslo, Sognsvannsveien 9, 0372 Oslo, Norway; 5 Department of Pharmacology and Toxicology, University of Toronto, 1 King’s College Circle, Toronto, ON M5S 1A8, Canada; 6 Howard Hughes Medical Institute, Department of Medicine and Department of Chemistry, The University of Chicago, Chicago, IL 60637, USA; 7 Lead contact

## Abstract

The regulation of type I interferon signaling is crucial for precisely tuning the innate immune response to combat pathogen invasions, fight cancer, and prevent autoimmune diseases. PARP7, a mono-ADP-ribosyltransferase also called TiPARP (tetrachlorodibenzo-p-dioxin [TCDD]-inducible PARP), is reported to inhibit the production of type I interferons. Here, we find that PARP7 suppresses type I interferon signaling instead of interferon production. PARP7 ADP-ribosylates and promotes the ubiquitination of signal transducer and activator of transcription 1 (STAT1) and STAT2, which recruits p62 to promote the degradation of STAT1 and STAT2 through autophagy. By reducing STAT1 and STAT2 levels, PARP7 decreases type I interferon signaling. We further show that the inhibition of PARP7 promotes type I interferon signaling and relieves experimental autoimmune encephalomyelitis (EAE) symptoms in mice. Our findings revealed a molecular mechanism via which PARP7 suppresses type I interferon signaling, offering insights into the immune-modulatory function of PARP7 and suggesting PARP7 inhibition as a potential treatment strategy for multiple sclerosis.

## In brief

The regulation of type I interferon signaling is essential for fine-tuning innate immunity against infections, cancer, and autoimmunity. Xu et al. uncover how PARP7 suppresses this pathway through mono-ADP-ribosylation of STAT1/STAT2, leading to their degradation. Inhibiting PARP7 restores interferon activity, offering a potential therapeutic approach for multiple sclerosis.

## Graphical Abstract

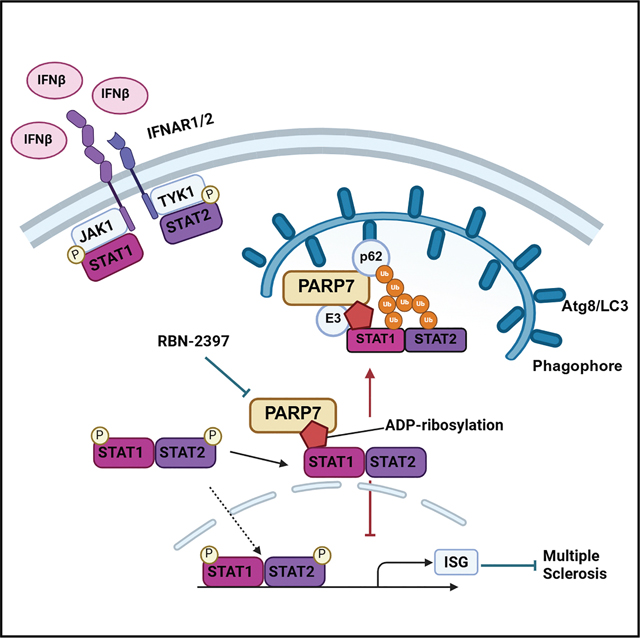

## INTRODUCTION

Type I interferons (IFN-Is) constitute a diverse group of cytokines activated in response to encounters with abnormal cytosolic nucleic acids.^1^ Potential sources of these nucleic acids include pathogens like viruses and bacteria, as well as abnormal endogenous nucleic acids in cancer cells.^2–4^ Recognition of these nucleic acids occurs through pattern recognition receptors (PRRs),^5,6^ such as the cGAS-STING pathway^7^ or the retinoic acid-inducible gene I (RIG-I) pathway.^8^ Both pathways lead to TBK1 phosphorylation and activation of IFN regulatory factor 3 (IRF3), which initiates IFN-I transcription.^7,9^ Secreted IFN-Is are then detected by IFN receptors on target cells, leading to the phosphorylation of Janus kinases (JAKs).^7,9,10^ Phosphorylated JAKs induce dimerization of signal transducer and activator of transcription 1 (STAT1) and STAT2, which enter the nucleus and trigger the transcription of IFN-stimulated genes (ISGs), promoting the innate or adaptive immune responses.^10–12^ Prior research underscores the crucial role of IFN-I signaling in anti-pathogen and anti-tumor immune responses.^2,3,13^ Dysregulation of IFN-I signaling contributes to autoimmune and inflammatory disorders.^14,15^ Therefore, understanding the regulatory mechanisms that control IFN-I production and signaling is important and may provide new strategies to treat human diseases in which dysregulation of the IFN-I signaling pathway is involved.

ADP-ribosylation, a protein post-translational modification, is the transfer of the ADP-ribosyl moiety of NAD^+^ to target proteins with concurrent release of nicotinamide.^16^ The diphtheria toxin-like ADP-ribosyltransferases, also called the ARTD or PARP family, are enzymes that catalyze ADP-ribosylation.^17,18^ Members of the PARP family are either poly(ADP-ribose) polymerases or mono(ADP-ribose) transferases.^18,19^ The functions of mono (ADP-ribose) transferases have been little understood compared to those of poly(ADP-ribose) polymerases, yet they play important roles in diverse biological processes,^20^ including viral transcription, protein synthesis, and modulation of GTPase activity.^21–23^

PARP7, also known as tetrachlorodibenzo-p-dioxin (TCDD)-inducible PARP (TIPARP), a mono-ADP-ribosyl transferase, has been identified as a suppressor of IFN-I production by ADP-ribosylation and inhibition of TBK1^2^ or nuclear factor κB (NF-κB).^13^ These findings highlight PARP7’s important role in regulating immune signaling pathways and suggest that PARP7 inhibitors could be useful for treating cancer and viral infections.

While substantial evidence supports PARP7’s role in regulating IFN-I production,^2^ there is also evidence suggesting that PARP7 may also regulate the downstream IFN signaling pathway,^3^ but it has not been widely recognized, and the mechanism through which this occurs is completely unknown. Here, we aim to investigate the mechanism of PARP7 regulation of the downstream JAK-STAT pathway. Interestingly, we found that PARP7 ADP-ribosylates STAT1/STAT2 and promotes their degradation through ubiquitination and p62-dependent autophagy. Inhibiting PARP7 increases IFN-I signaling and decreases experimental autoimmune encephalomyelitis (EAE) symptoms in a mouse model of multiple sclerosis (MS). These findings offer crucial insights into the physiological roles of PARP7 and reveal potential applications of PARP7 inhibitors in MS treatment.

## RESULTS

### PARP7 suppresses IFN-1 signaling by reducing STAT1/STAT2 protein levels

**Figure 1.**
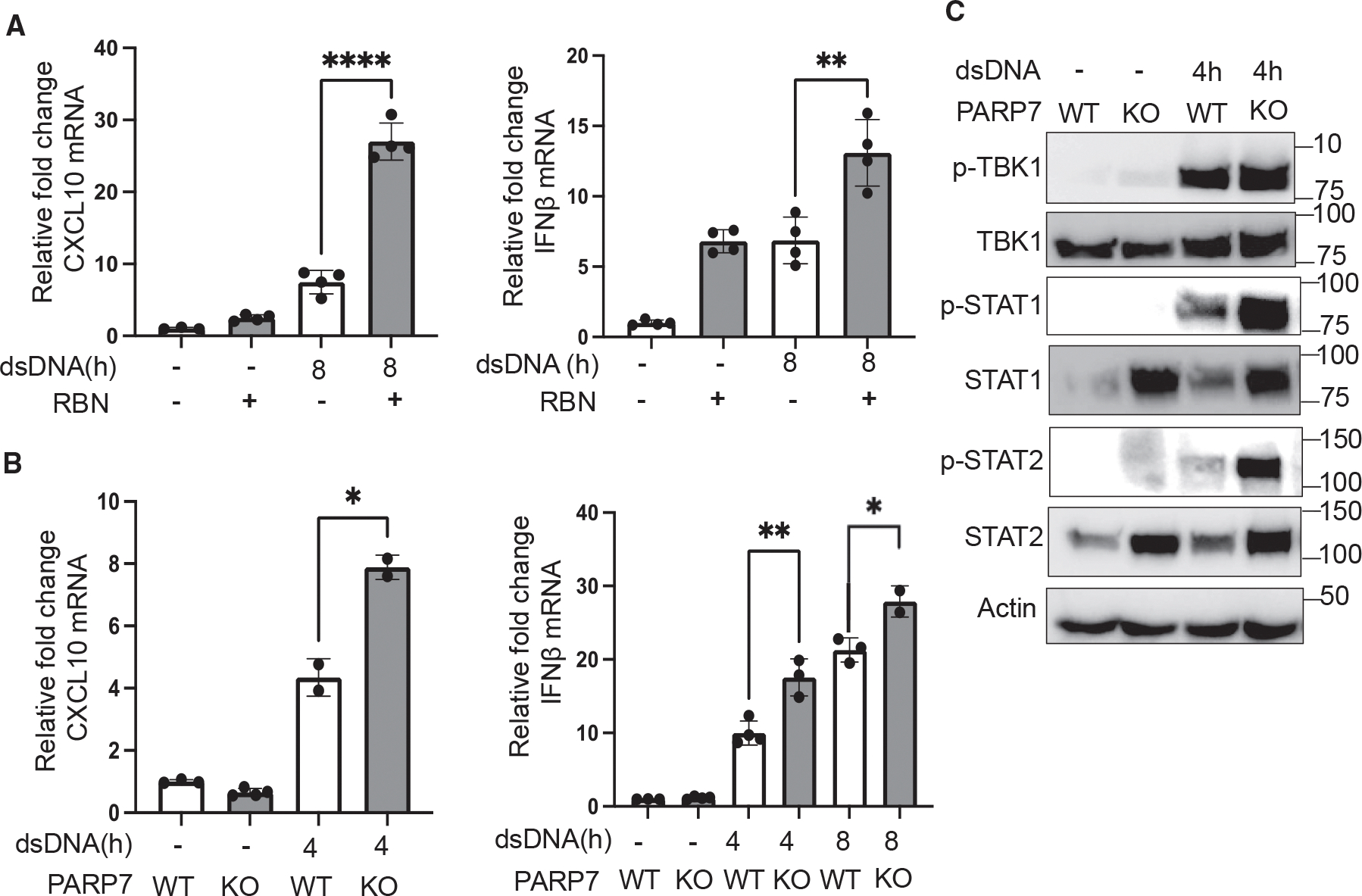
PARP7 deficiency and inhibition increase IFN-I signaling with dsDNA treatment (A) The expression level of the CXCL10 (left) and IFNβ (right) genes (measured by qPCR) with or without the PARP7 inhibitor RBN-2397 (RBN; 500 nM) after dsDNA (2.5 ng/mL) treatment in MEF *Parp7*^+/+^ cells. Statistical significance was determined using an unpaired t test (data presented as mean ± SEM, n = 4, **p < 0.005, ****p < 0.0001). (B) Gene expression levels of CXCL10 and IFNβ (measured by qPCR) with or without dsDNA (2.5 ng/mL) treatment in *Parp7*^+/+^ and *Parp7*^−/−^ MEF cells. Statistical significance was determined using an unpaired t test (data presented as mean ± SEM, n = 2 or 4, *p < 0.05, **p < 0.01). (C) Immunoblot analysis of type I IFN signaling pathway in *Parp7*^+/+^ and *Parp7*^−/−^ MEF cells with or without dsDNA (2.5 ng/mL) treatment. All experiments were repeated three times. See also Figure S1.

Given the prior report that PAPP7 suppresses IFN-I production, we first confirmed that PARP7 regulates IFNβ production and IFN-I signaling with the transfection of poly(dA:dT) (double-stranded DNA [dsDNA]) in PARP7 wild-type (WT) and knockout (KO) mouse embryonic fibroblast (MEF) cells (Figures S1A and S1B). Consistent with previous reports, PARP7 inhibition or deficiency significantly upregulated *IFNβ* and *CXCL10* gene transcription (Figures 1A and 1B). The *CXCL10* gene is the downstream gene of IFN-I signaling, and the levels of *IFNβ* and *CXCL10* indicate IFN-I production and IFN-I signaling, respectively. However, different from prior literature (which used vesicular stomatitis virus or 5’-triphosphate RNA treatment),^2^ PARP7 deficiency did not dramatically affect phosphorylated (p)-TBK1 and TBK1 levels with double-strand DNA (dsDNA) transfection (Figure 1C). In contrast, PARP7 deficiency dramatically increased the protein levels of STAT1 and STAT2, as well as their phosphorylated forms (p-STAT1 and p-STAT2), with dsDNA treatment (Figure 1C). In particular, the levels of STAT1 and STAT2 were increased by PARP7 KO even without dsDNA treatment. These results suggest that PARP7 may regulate STAT1/STAT2 and thus IFN-I signaling instead of (or in addition to) IFN production.

**Figure 2.**
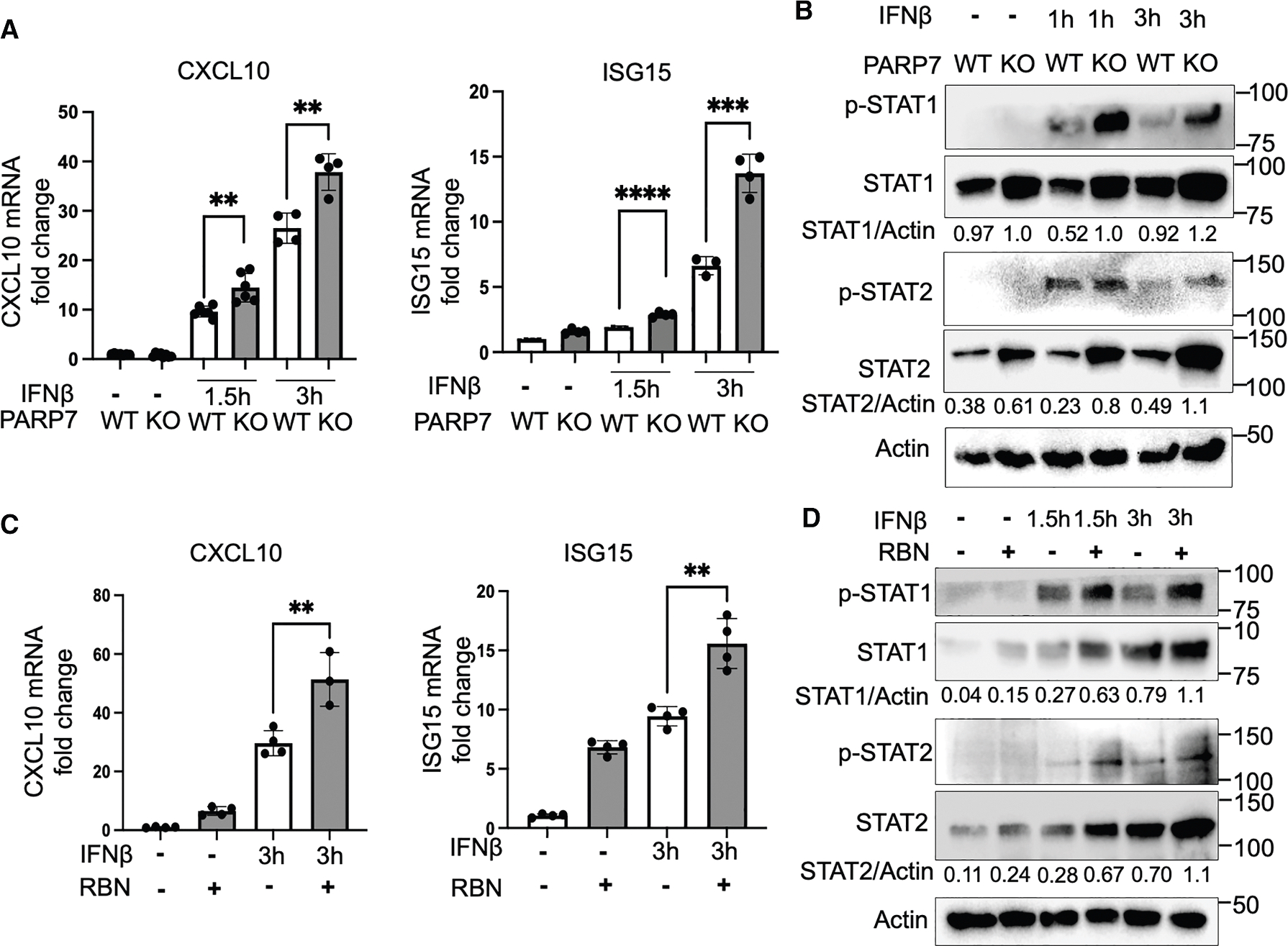
PARP7 knockout or inhibition promotes IFN-I signaling initiated by IFNβ treatment (A) The mRNA levels of CXCL10 and ISG15 (measured by qPCR) under IFNβ (2 ng/mL) treatment in MEF *Parp7*^+/+^ and *Parp7*^−/−^ cells. Statistical significance was determined using an unpaired t test (*p < 0.05, **p < 0.01, ***p < 0.001). Error bars represent standard error, *n* = 4. (B) Immunoblot analysis of IFN-I signaling pathway in MEF *Parp7*^+/+^ and *Parp7*^−/−^ cells with or without IFNβ (2 ng/mL) treatment. (C) The mRNA levels of CXCL10 and ISG15 (measured by qPCR) under 3 h of IFNβ (2 ng/mL) treatment in MEF *Parp7*^+/+^ cells with or without RBN-2397 (500 nM). Statistical significance was determined using an unpaired t test (**p < 0.01). Error bars represent standard error, *n* = 3. (D) Immunoblot analysis of IFN-I signaling pathway in *Parp7*^+/+^ MEF cells under IFNβ (2 ng/mL) treatment with or without RBN-2397 (500 nM). All experiments were done in triplicates. See also Figure S1.

To test whether PARP7 directly regulates IFN-I signaling pathway, we treated both *Parp7*^+/+^ and *Parp7*^−/−^ MEF cells with IFNβ and examined the expression of the genes downstream of the JAK-STAT pathway, *CXCL10* and *ISG15*. PARP7 deficiency resulted in the upregulation of both *CXCL10* and *ISG15* mRNA levels after IFNβ treatment (Figure 2A). The levels of p-STAT1/p-STAT2 and STAT1/STAT2 were higher in *Parp7*^−/−^ MEF cells before and after IFNβ treatment, compared with *Parp7*^+/+^ MEF cells (Figure 2B). The differences in p-STAT1/p-STAT2 levels are likely due to the differences in STAT1/STAT2 protein levels. Furthermore, PARP7 inhibitor RBN-2397 (RBN) also upregulated *CXCL10* and *ISG15* genes after 1.5 and 3 h of IFNβ treatment and stabilized the p-STAT1/p-STAT2 and STAT1/STAT2 levels (Figures 2C and 2D), suggesting that the regulation of STAT1/STAT2 by PARP7 depends on its catalytic activity. Since IFNβ signaling does not go through STING-TBK1 or NF-κB, these results suggest that PARP7 can directly regulate the JAK-STAT pathway.

### PARP7 ADP-ribosylates STAT1/STAT2

**Figure 3.**
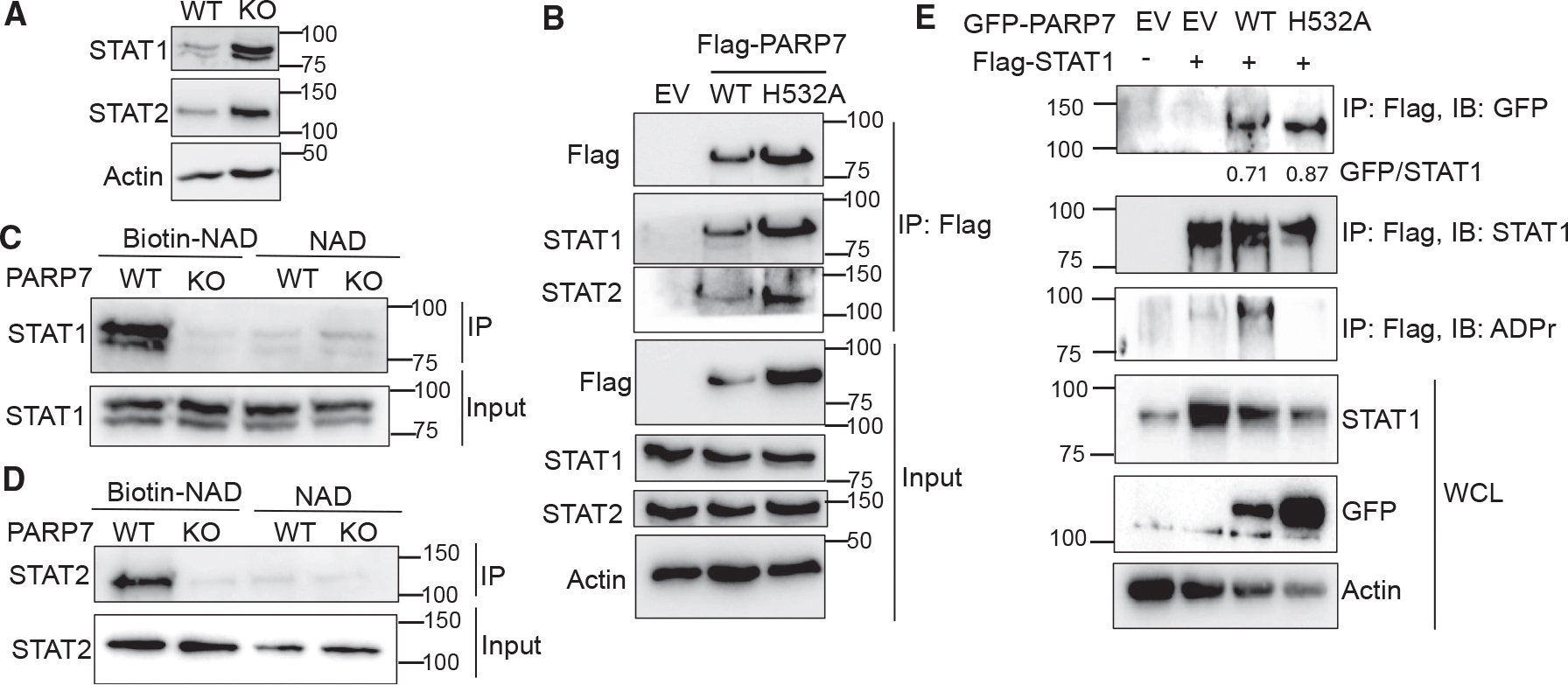
PARP7 ADP-ribosylates STAT1 and STAT2 (A) Immunoblot analysis of STAT1 and STAT2 protein levels in PARP7 WT and KO MEF cells. The experiment was repeated twice. (B) Co-immunoprecipitation (coIP) analysis showing that STAT1 and STAT2 interact with PARP7. FLAG-PARP7 was ectopically expressed in HEK293T cells. FLAG-PARP7 was immunoprecipitated and then blotted for endogenous STAT1 and STAT2. The experiment was repeated twice. (C and D) Immunoblot analysis of ADP-ribosylated STAT1 (C) and STAT2 (D) in PARP7 WT and KO MEF cells. Cells were lysed in the presence of biotin-NAD^+^ or NAD^+^. Proteins that were ADP-ribosylated by biotin-NAD^+^ were pulled down with streptavidin beads and then blotted for STAT1 or STAT2. Samples treated with NAD^+^ were used as a negative control. Both experiments were repeated three times. (E) PARP7 WT, but not the H532A mutant, ADP-ribosylates STAT1. GFP-tagged empty vector (EV), WT PARP7, or the H532 mutant were co-transfected with FLAG-STAT1 into HEK293T cells. FLAG-STAT1 was pulled down to detect interaction with PARP7 (blotted with GFP antibody) and ADP-ribosylation (blotted with ADP-ribose antibody). The experiment was repeated twice. See also Figure S2.

PARP7 is known to ADP-ribosylate and downregulate transcription factors like HIF1α. Since STAT1 and STAT2 are transcription factors and their protein levels are both downregulated by PARP7, we examined whether they are substrates of PARP7. Both STAT1 and STAT2 protein levels were higher in *Parp7*^−/−^ MEF cells than in *Parp7*^+/+^ MEF cells (Figure 3A). We confirmed that PARP7 interacted with STAT1/STAT2. The FLAG-tagged WT and catalytic dead H532A mutant of PARP7 co-immunoprecipitated endogenous STAT1 and STAT2 (Figure 3B). Thus, PARP7 interacts with STAT1/STAT2 independent of PARP7’s catalytic activity.

We then checked if STAT1 and STAT2 are ADP-ribosylated by PARP7. In *Parp7*^+/+^ and *Parp7*^−/−^ MEF cell lysates, we added biotin-NAD^+^ to allow PARP7 to biotin-ADP-ribosylate its substrate proteins. We then used streptavidin beads to pull down the labeled substrate proteins and blotted for STAT1 and STAT2. The result showed that STAT1/STAT2 were pulled down in *Parp7*^+/+^ cell lysates treated with biotin-NAD^+^ but not in cell lysates treated with NAD^+^ or in *Parp7*^−/−^ cell lysates (Figures 3C and 3D). These results suggest that PARP7 ADP-ribosylates STAT1 and STAT2. To further confirm this with a different approach, HEK293T cells were co-transfected with FLAG-empty vector (EV) or FLAG-STAT1 and GFP-EV, GFP-PARP7 WT, or GFP-PARP7 H532A.^24^ We immunoprecipitated FLAG-STAT1 and blotted for GFP-PARP7, STAT1, and STAT1 ADP-ribosylation. Both the PARP7 WT and H532A mutant co-immunoprecipitated with FLAG-STAT1, confirming that their interaction is independent of the catalytic activity of PARP7 (Figure 3E). PARP7, but not the PARP7 H532A inactive mutant, ADP-ribosylated FLAG-STAT1 (Figure 3E). To directly validate *in vitro* that PARP7 ADP-ribosylates STAT1 and STAT2, we incubated purified PARP7 and STAT1 or STAT2 with or without NAD^+^ (Figures S2A–S2C). We found that only with the presence of PARP7 and NAD^+^ can STAT1 and STAT2 be ADP-ribosylated (Figure S2C). The data further support that PARP7 ADP-ribosylates STAT1.

### PARP7 recruits STAT1/STAT2 to PARP7 foci

**Figure 4.**
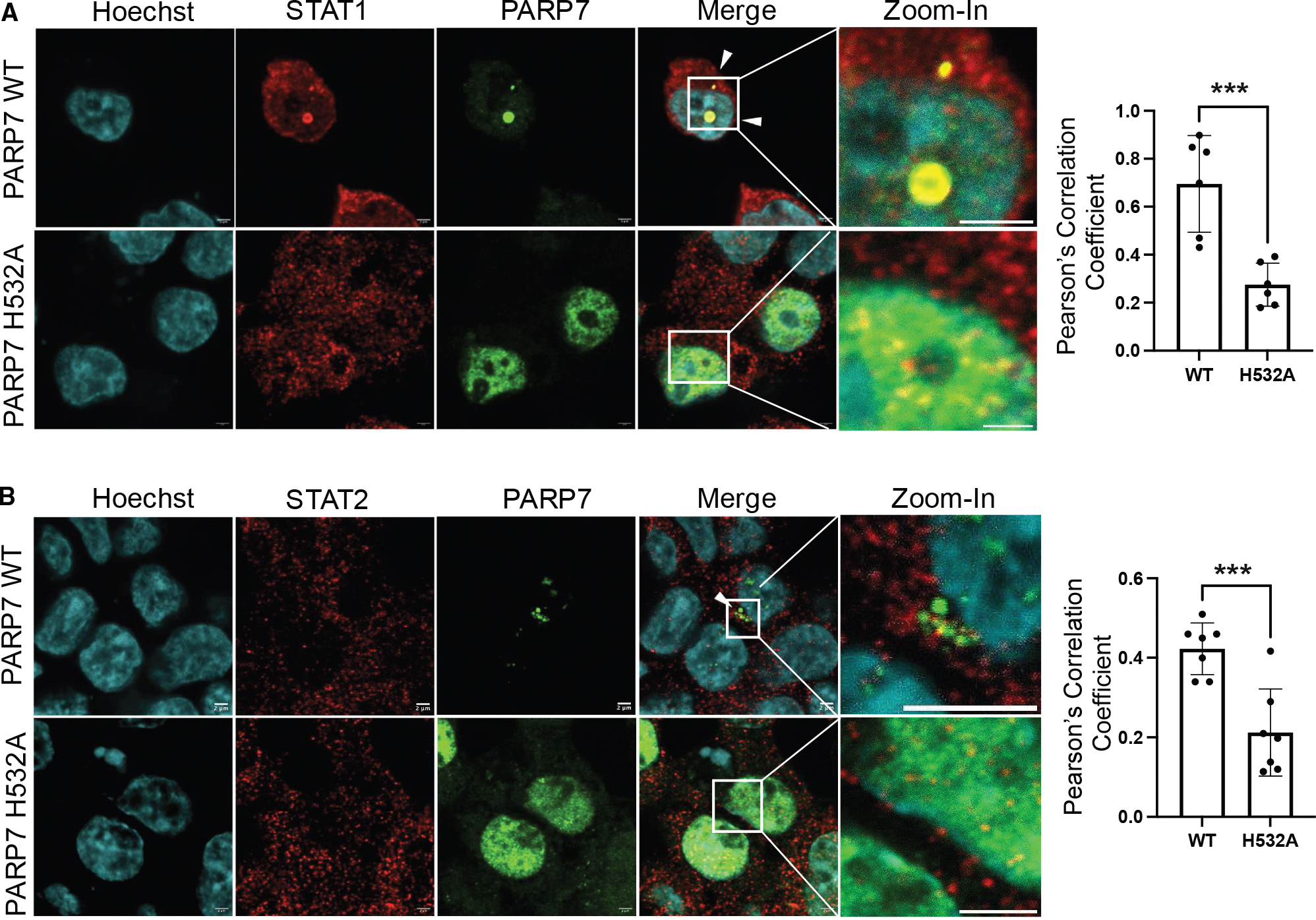
PARP7 forms foci and recruits STAT1 and STAT2 (A) HEK293T cells were transfected with FLAG-WT or H532A mutant PARP7 overnight. FLAG-PARP7 was detected by immunofluorescence with anti-FLAG (green) antibodies. Endogenous STAT1 was detected by immunofluorescence with Alexa Flour 647. Nuclei were stained with Hoechst stain (blue) (scale bar: 2 μm). Right: quantification of STAT1 and PARP7 co-localization using Pearson’s correlation coefficient. Data are presented as mean ± SD. ***p < 0.001, unpaired t test. (B) HEK293T cells were transfected with FLAG-WT or H532A mutant PARP7 overnight. FLAG-PARP7 was detected by immunofluorescence with anti-FLAG (green) antibodies. Endogenous STAT2 was detected by immunofluorescence with Alexa Flour 647. Nuclei were stained with Hoechst stain (blue) (scale bar: 2 μm.). Right: quantification of STAT2 and PARP7 co-localization using Pearson’s correlation coefficient. Data are presented as mean ± SD. ***p < 0.001, unpaired t test. All experiments were repeated twice. See also Figure S3.

PARP7 is known to form nuclear foci and recruit transcription factors to the foci to promote their degradation.^5^ Thus, we investigated whether STAT1 and STAT2 are also recruited to PARP7 foci in cells. We ectopically expressed FLAG-STAT1 and FLAG-STAT2 in HEK293T cells.^25^ In the absence of PARP7 expression, FLAG-STAT1 was mainly distributed in the nucleus, but there was also some cytosolic localization, while FLAG-STAT2 primarily localized in the cytosol (Figures 4A and 4B). Upon expression, PARP7 formed foci that co-localized with endogenous STAT1 and STAT2 (Figures 4A and 4B). Although PARP7 can form nuclear foci as previously described,^20^ we noticed here that PARP7 can also form foci in the cytosol. Intriguingly, the catalytic mutant of PARP7 (H532A) failed to form foci that co-localized with STAT1 or STAT2 (Figures 4A and 4B). To further prove the idea, we also co-overexpressed GFP-PARP7 (WT and H532A) and FLAG-STAT1/FLAG-STAT2 in HEK293T cells (Figures S3A and S3B). Immunofluorescence results show that WT PARP7 formed cytosolic foci in HEK293T cells and co-localized with STAT1 and STAT2, while its catalytic mutant did not (Figures S3A and S3B). The data suggest that PARP7 can form foci in the cytosol in an ADP-ribosylation-dependent manner and recruit STAT1 and STAT2 to the foci, similar to the formation of PARP7 nuclear foci and recruitment of HIF1α.^5^

### PARP7 promotes STAT1/STAT2 ubiquitination and lysosomal degradation

**Figure 5.**
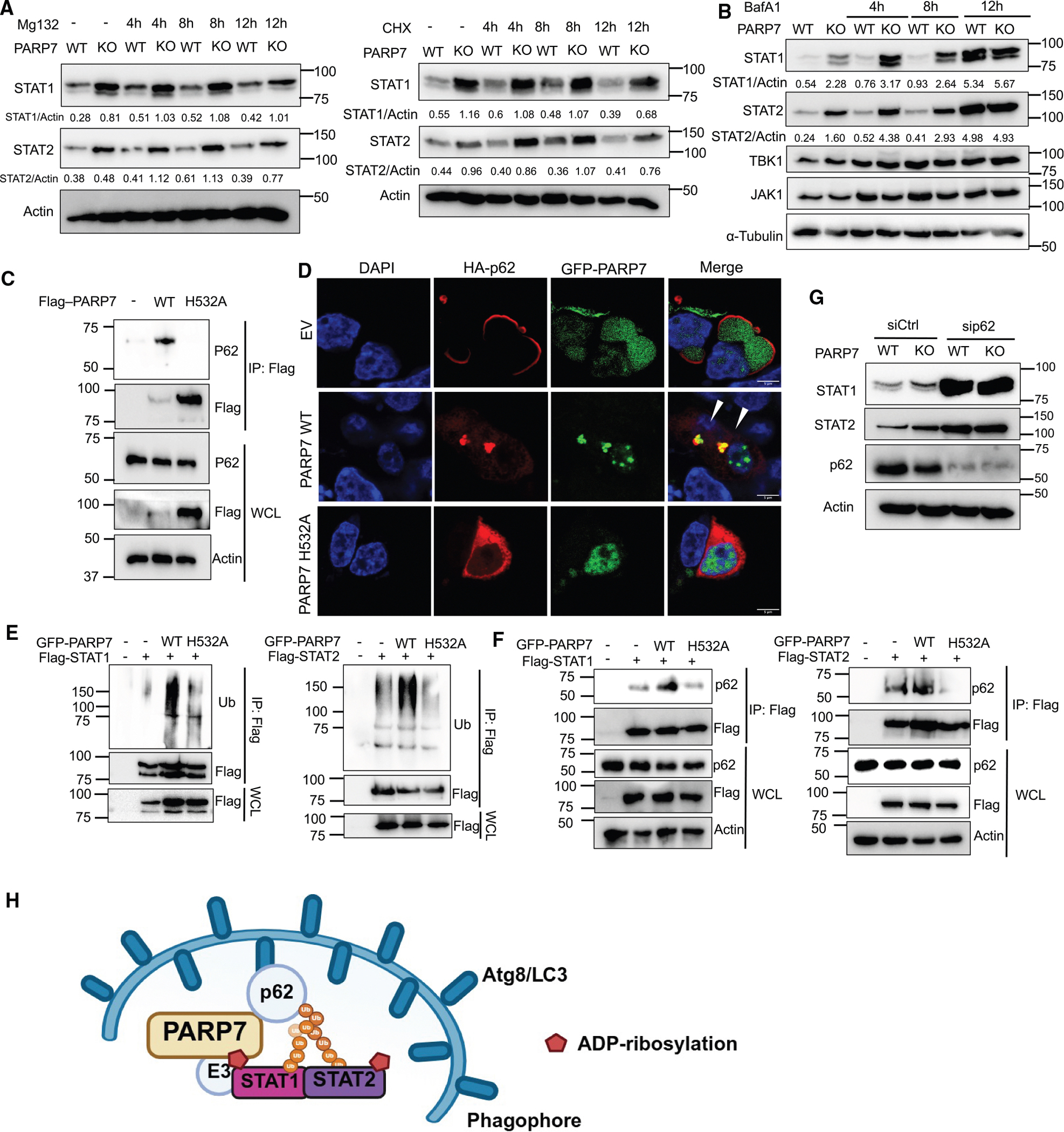
PARP7 promotes STAT1 and STAT2 degradation through autophagy by recruiting p62 (A) Western blot analysis of STAT1 and STAT2 in PARP7 WT or KO MEF cells after treatment of MG132 at 20 μМ (left) and cycloheximide (CHX) at 50 μМ (right). The experiment was repeated three times. (B) Western blot analysis of STAT1 and STAT2 in PARP7 WT or KO MEF cells after bafilomycin A1 treatment at 5 μМ. The experiment was repeated three times. (C) coIP analysis of p62 and PARP7 WT or H532A catalytic mutant. FLAG-tagged PARP7 WT and H532A mutant were transfected into HEK293T cells, and coIP was performed to detect interactions between p62 and PARP7 WT or H532A mutant. The experiment was repeated three times. (D) Co-localization of hemagglutinin (HA)-tagged p62 with GFP-tagged PARP7 WT or H532A mutant in HEK293T cells. HA-p62 was detected by immunofluorescence with anti-HA (red) antibody. Nuclei were stained with Hoechst stain (scale bar: 5 μm). The experiment was repeated twice. (E) PARP7 promotes STAT1 and STAT2 ubiquitination. HEK293T cells were co-transfected with FLAG-STAT1 (left) or FLAG-STAT2 (right) and GFP-tagged PARP7 WT or H532A mutant. STAT1 or STAT2 was immunoprecipitated by anti-FLAG affinity resins, and the ubiquitination of STAT1 or STAT2 was analyzed by western blot. The experiment repeated twice. (F) PARP7 promotes p62 interaction with STAT1 and STAT2. HEK293T cells were co-transfected with FLAG-STAT1 (left) or FLAG-STAT2 (right) and GFP-tagged PARP7 WT or H532A mutant. STAT1 or STAT2 was immunoprecipitated by anti-FLAG affinity resin and blotted for p62. The experiment was repeated twice. (G) p62 knockdown diminishes the difference in STAT1 and STAT2 levels between PARP7 WT and KO MEF cells. STAT1 and STAT2 protein levels in PARP7 WT and KO MEF cells with or without p62 knockdown were analyzed by western blots. The experiment was repeated twice. (H) Proposed model depicting the negative regulation of STAT1 and STAT2 by PARP7. PARP7 binds and ADP-ribosylates STAT1 and STAT2. The ADP-ribosylation recruits E3 ubiquitin ligases, leading to the ubiquitination of STAT1 and STAT2. The ubiquitinated STAT1 and STAT2 then recruits p62, leading to autophagy-mediated degradation. Created with BioRender.com. See also Figures S4–S6.

Given that PARP7 ADP-ribosylates STAT1 and STAT2, decreases their protein levels, and recruits them to the PARP7 nuclear foci, we hypothesized that the mechanism of STAT1/STAT2 regulation by PARP7 may be similar to the regulation of HIF1α by PARP7, in which PARP7 forms nuclear foci to recruit E3 ligase HUWE1 to ubiquitinate HIF1α and promotes its proteasomal degradation.^20^ We thus treated both *Parp7*^+/+^ and *Parp7*^−/−^ MEF cells with cycloheximide (CHX; a protein synthesis inhibitor) or MG132 (a proteasome inhibitor) to see whether the protein levels of STAT1/STAT2 would be affected. Neither CHX nor MG132 affected the suppressive effect of PARP7 on STAT1/STAT2 protein levels (Figure 5A). The lack of effect of CHX was expected, as our model is that PARP7 affects STAT1/STAT2 protein degradation, not synthesis. However, the lack of effect of MG132 was surprising. Instead, we found that treating the cells with bafilomycin A1 (BafA1), a lysosome inhibitor, strongly diminished the difference in STAT1/STAT2 protein levels in *Parp7*^+/+^ and *Parp7*^−/−^ MEF cells (Figure 5B). These results suggest that PARP7 promotes the degradation of STAT1/STAT2 through lysosomal degradation. Combined treatment with RBN and BafA1 further stabilized STAT1/STAT2 and p-STAT1/p-STAT2 protein levels compared to individual treatments, suggesting that simultaneous inhibition of TiAPRP and autophagy more effectively restores IFN-I signaling (Figure S5A).

To understand how PARP7 promotes the lysosomal degradation of STAT1/STAT2, we investigated the role of p62, an autophagy adaptor.^20^ In autophagy, p62 serves as a cargo receptor, with its involvement mediated by the LC3-interacting region (LIR) linking it to LC3 on autophagosomes and the ubiquitin-associated (UBA) domain that binds to ubiquitinated cargos. p62 was identified as a potential interactor of PARP7 in previous interactome studies.^5^ In HEK293T cells, we overexpressed FLAG-EV, FLAG-PARP7 WT, or FLAG-PARP7 H532A. Co-immunoprecipitation revealed that p62 interacted exclusively with WT PARP7 but not the H532A catalytic mutant (Figure 5C). This indicates that the binding of p62 to PARP7 hinges on the ADP-ribosylation activity of PARP7. Imaging studies further demonstrated the recruitment of p62 to PARP7 cytosolic bodies but not PARP7 nuclear bodies. Notably, the H532A mutant of PARP7 failed to recruit p62 (Figure 5D). Additionally, FLAG-PARP7 WT also co-localized with endogenous p62 in HEK293T cells in the cytosol (Figure S4). However, FLAG-PARP7 H532A did not form cytosolic foci or recruit p62 in the cytosol (Figure S4). Considering that p62 contains a UBA domain, we explored whether PARP7 enhances the interaction between p62 and STAT1/STAT2 by promoting the ubiquitination of STAT1/STAT2. Indeed, overexpressing PARP7 WT, but not the H532A mutant, facilitated the ubiquitination of STAT1 and STAT2 in HEK293T cells (Figure 5E) and promoted the interaction between p62 and FLAG-STAT1 and FLAG-STAT2 (Figure 5F).

In MEF cells, knocking down p62 resulted in higher levels of endogenous STAT1 and STAT2, eliminating the difference between *Parp7*^+/+^ and *Parp7*^−/−^ MEF cells (Figure 5G). Knocking down p62 in MEF cells also restored IFN-I signaling, as indicated by higher p-STAT1 and p-STAT2 levels compared to the no-treatment control (Figure S5B). To further confirm the mechanism in MEF cells, we performed endogenous immunoprecipitation (IP) to pull down STAT1 and STAT2 (Figures S6A and S6B). We found that PARP7 interacted with STAT1 and STAT2 (Figures S6A and S6B). WT PARP7 promoted the ADP-ribosylation and ubiquitination of STAT1 and STAT2 and their interaction with p62. These findings are consistent with the mechanism we found in HEK293T cells (Figure 5).

Collectively, our data demonstrated that PARP7 directly suppresses IFN-I signaling through foci formation and ADP-ribosylation of STAT1 and STAT2, promoting their ubiquitination and p62-dependent autophagic degradation (Figure 5H).

### PARP7 inhibition/deficiency protects mice in EAE model

The finding that PARP7 directly suppresses IFN-I signaling suggests that PARP7 inhibitors could potentially be beneficial to treat human diseases. For example, the first FDA-approved treatment for MS was IFNβ.^26^ Since PARP7 inhibition and deficiency can increase IFN-I signaling, we proceed to explore whether PARP7 is a potential therapeutic target for MS.

**Figure 6.**
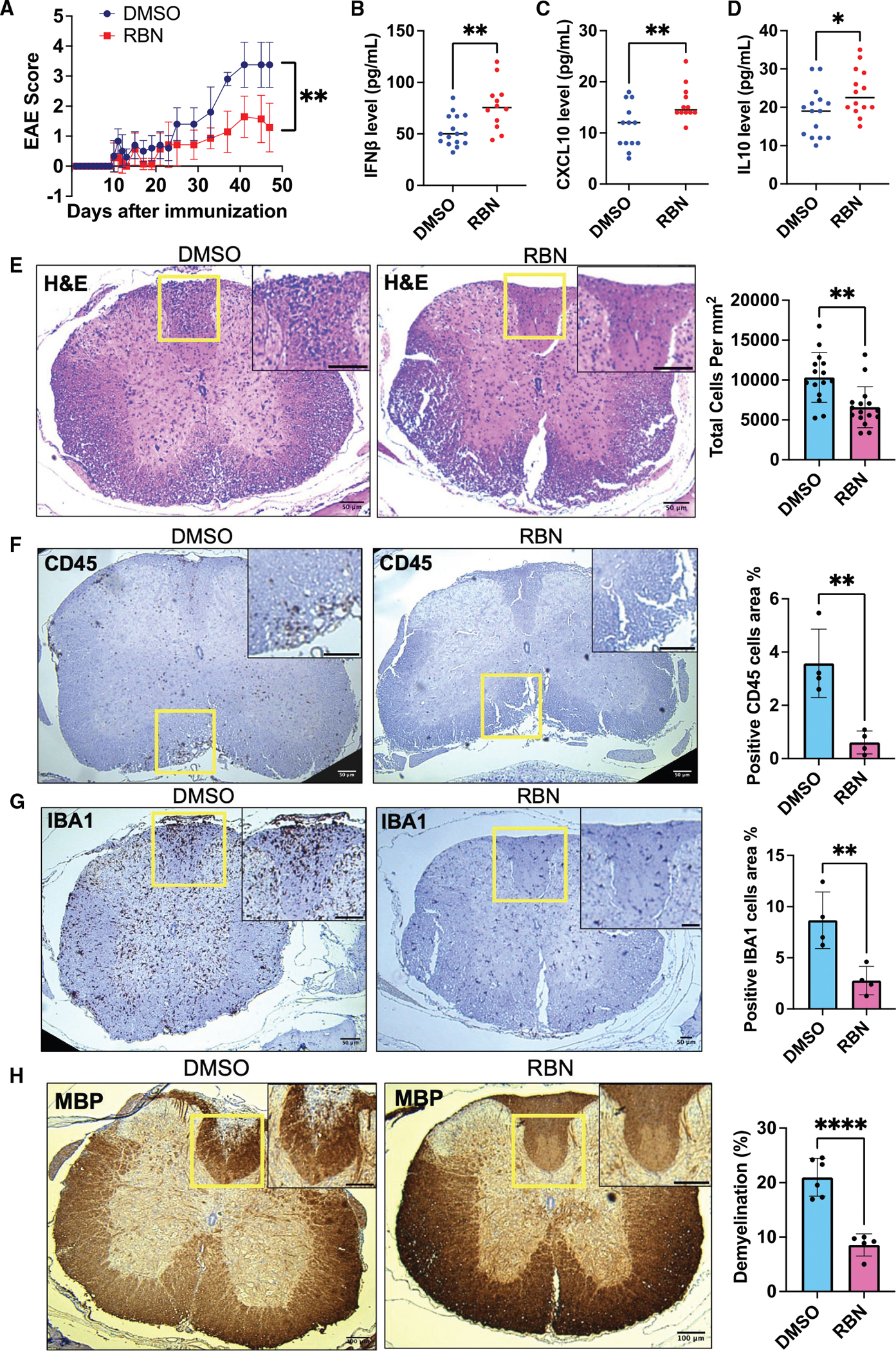
PARP7 inhibition attenuates EAE progression and neuroinflammation (A) Clinical scores of EAE in PARP7 WT mice treated with vehicle (DMSO) or RBN-2397 compound (RBN) over time following immunization. Representative data are shown. DMSO: *n* = 6; RBN: *n* = 7. Data are presented as mean ± SEM. Statistical analysis was performed using two-way ANOVA with Bonferroni’s post hoc test (**p < 0.005). Total three repeats were performed. (B) IFNβ levels in the serum of DMSO- or RBN-treated mice. Data are presented as mean ± SEM. Statistical analysis by unpaired two-tailed t test (**p < 0.005). (C) CXCL10 levels in the serum of DMSO- vs. RBN-treated mice. Data are presented as mean ± SEM. Statistical analysis by unpaired two-tailed t test (**p < 0.005). (D) IL-10 levels in the serum from DMSO- vs. RBN-treated mice. IL-10 was significantly elevated with RBN treatment. Data are presented as mean ± SEM. Statistical analysis by unpaired two-tailed t test (*p < 0.05). (E) H&E staining of spinal cord sections from DMSO- and RBN-treated mice. Representative images show reduced cellular infiltration in RBN-treated mice. Quantification of total cell density (cells/mm^2^) is shown to the right (mean ± SEM, **p < 0.01). Scale bars: 50 μm. (F) Immunohistochemical staining for CD45 (a pan-leukocyte marker) in spinal cords from DMSO- and RBN-treated mice. RBN groups show reduced immune cell infiltration. Quantification of CD45^+^ area (%) is shown (mean ± SEM, **p < 0.01). Scale bars: 50 μm. (G) Immunohistochemistry staining for IBA1, a marker of microglia and macrophages, in spinal cords from DMSO- and RBN-treated mice. Representative images show decreased activation and infiltration in RBN-treated mice. Quantification of IBA1^+^ area (%) is shown (mean ± SEM, **p < 0.01). Scale bars: 50 μm. (H) Immunohistochemistry staining for myelin in spinal cords from DMSO- and RBN-treated mice. RBN-treated mice showed improved myelin preservation compared to DMSO-treated mice. Quantification of demyelinated area (%) demonstrates significantly less demyelination in RBN-treated mice (mean ± SEM, ****p < 0.0001). Scale bars: 50 μm. See also Figures S1 and S7.

We first tested whether the PARP7 inhibitor RBN could be helpful for the treatment of MS in a mouse EAE model. We immunized C57BL/6 mice with a myelin oligodendrocyte glycoprotein (MOG) peptide (MOG35-55) along with pertussis toxin to induce EAE, a widely used mouse model for MS. The EAE-induced mice were administered intraperitoneally with either DMSO or the PARP7 inhibitor RBN every other day. As expected, the DMSO group developed severe clinical symptoms that were characterized by a gradual increase in the severity of paralysis. In contrast, the RBN-treated group had much milder clinical symptoms (Figure 6A).

**Figure 7.**
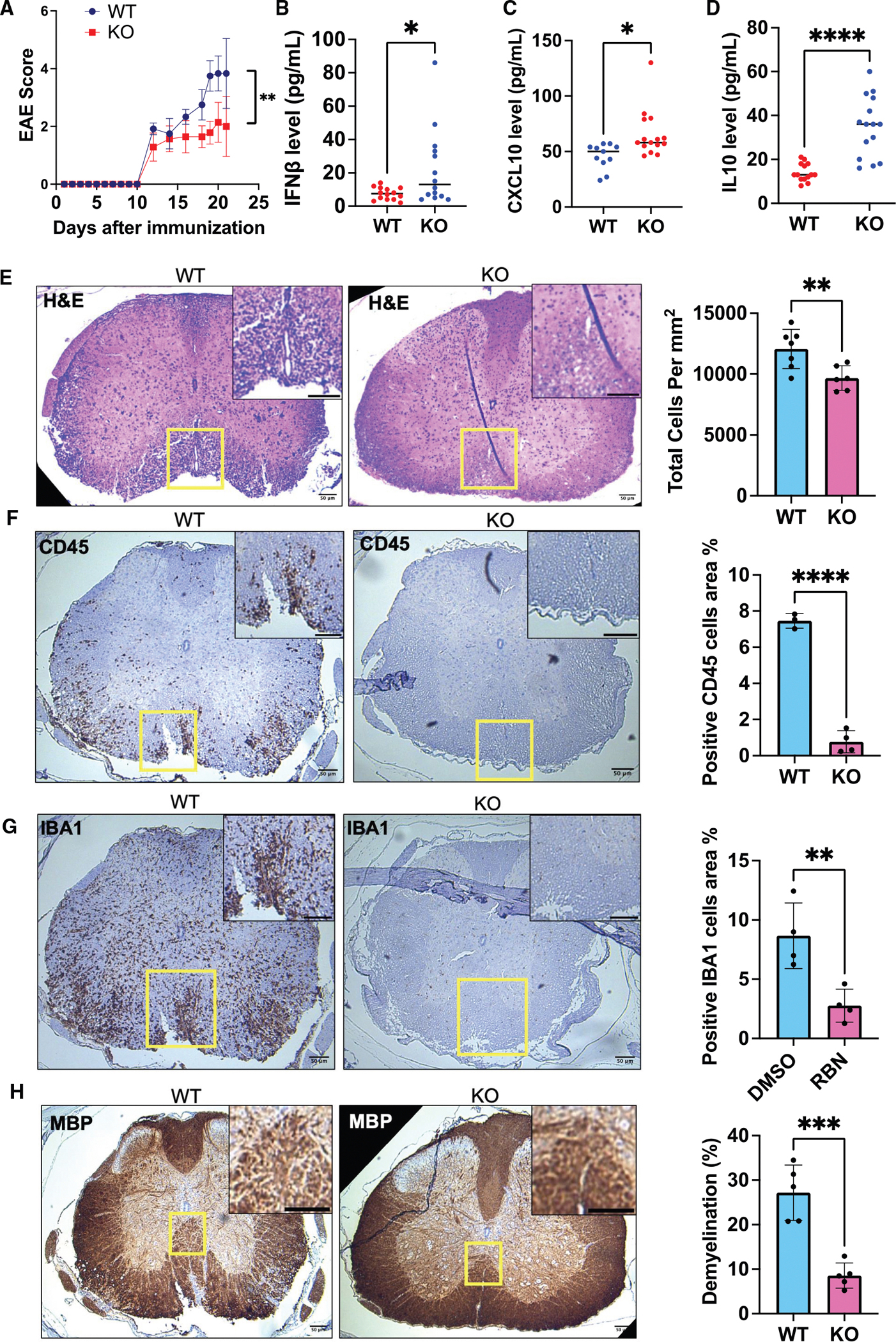
PARP7 deficiency attenuates EAE progression and neuroinflammation (A) Clinical scores of EAE in PARP7 wild-type (WT) and knockout (KO) mice over time following immunization. Representative data are shown. WT: *n* = 6; KO: *n* = 7. Data are presented as mean ± SEM. Statistical analysis was performed using two-way ANOVA with Bonferroni’s post hoc test (**p < 0.01). Two repeats were performed. (B) IFNβ levels in the serum of PARP7 WT vs. KO mice. Data are presented as mean ± SEM. Statistical analysis by unpaired two-tailed t test (*p < 0.05). (C) CXCL10 levels in the serum of PARP7 WT vs. KO mice. Data are presented as mean ± SEM. Statistical analysis by unpaired two-tailed t test (*p < 0.05). (D) IL-10 levels in the serum from WT vs. KO mice. IL-10 was significantly elevated in KO mice. Data are presented as mean ± SEM. Statistical analysis by unpaired two-tailed t test (****p < 0.0001). (E) H&E staining of spinal cord sections from WT and KO mice. Representative images show reduced cellular infiltration in KO mice. Quantification of total cell density (cells/mm^2^) is shown to the right (mean ± SEM, **p < 0.01). Scale bars: 50 μm. (F) Immunohistochemical staining for CD45 (a pan-leukocyte marker) in spinal cords from PARP7 WT and KO mice. KO groups show reduced immune cell infiltration. Quantification of CD45^+^ area (%) is shown (mean ± SEM, ****p < 0.0001). Scale bars: 50 μm. (G) Immunohistochemistry staining for IBA1, a marker of microglia and macrophages, in spinal cords from PARP7 WT and KO mice. Representative images show decreased activation and infiltration in KO mice. Quantification of IBA1^+^ area (%) is shown (mean ± SEM, **p < 0.01). Scale bars: 50 μm. (H) Immunohistochemistry staining for myelin in spinal cords from WT and KO mice. KO mice showed improved myelin preservation compared to WT. Quantification of demyelinated area (%) demonstrates significantly less demyelination in both KO and RBN-treated mice (mean ± SEM, ***p < 0.001). Scale bars: 50 μm. See also Figures S1 and S7.

We then generated PARP7 KO C57BL/6 mice to compare the MS symptoms in PARP7 WT and KO mice (Figures S1C–S1E). We performed the EAE model using PARP7 WT and KO mice and found that the KO mice had much milder clinical symptoms than WT mice (Figure 7A).

To validate that IFNβ production and signaling were affected by PARP7 KO or inhibition, as we observed in cellular studies, we checked IFNβ and CXCL10 levels in the mouse serum in the EAE model (Figures 6B, 6C, 7B, and 7C). Consistent with cellular experiments, there were higher IFNβ and CXCL10 levels in PARP7 KO and RBN-treated mice compared to PARP7 WT and DMSO-treated mice (Figures 6B, 6C, 7B, and 7C).^27^ We also checked interleukin (IL)-10 levels in the EAE mice. Higher levels of IL-10 were detected in PARP7 KO and RBN-treated mice than in PARP7 WT and DMSO-treated mice (Figures 6D and 7D). Higher total STAT1 and p-STAT1 levels of homogenized spinal cords of RBN-treated mice and PARP7 KO mice (Figure S7) also indicated higher IFN-I signaling, consistent with cellular data. Hematoxylin and eosin (H&E) staining of the spinal cords at similar positions showed more inflammatory infiltrates mainly localized around the marginal zone in the DMSO-treated and WT mice compared to the RBN-treated and KO mice (Figures 6E and 7E). The increase of immune cell influx into the central nervous system (CNS) triggers inflammation, myelin damage, and axonal damage. Staining for CD45, a pan-leukocyte marker, revealed substantially less immune cell infiltration in the spinal cords of KO and RBN-treated animals (Figures 7F and 8F). Additionally, staining for IBA1, a marker of microglia and macrophages, showed reduced activation and infiltration in both experimental groups (Figures 6G and 7G), indicating attenuated innate immune activation within the CNS. Consistently, based on the myelin basic protein (MBP) staining, demyelination was more severe in the spinal cord of PARP7 WT and DMSO-treated EAE mice than in that of PARP7 KO and RBN-treated mice (Figures 6H and 7H). These data collectively suggest that PARP7 inhibition or deficiency could relieve EAE symptoms.

## DISCUSSION

IFN-I signaling plays pivotal roles in combating pathogens, preventing cancer, and the onset of autoimmune diseases.^13,15,28^ Our work here establishes a previously unknown regulatory function of PARP7 on STAT1 and STAT2, two crucial transcription factors mediating IFN-I signaling (Figure 5H). Our data show that PARP7 binds to and ADP-ribosylates STAT1 and STAT2. The ADP-ribosylation promotes STAT1 and STAT2 ubiquitination, recruitment of p62, and subsequent degradation through autophagy. This is a new regulatory mechanism of the IFN-I signaling pathway that bears important physiological significance.

A previous report shows that PARP7 ADP-ribosylates and negatively regulates TBK1 under infection by vesicular stomatitis virus or 3pRNA treatment, thus affecting the production of IFNs.^2^ Other studies have suggested that PARP7 may regulate other pathways related to IFN production.^3,13,29,30^ For example, PARP7 inhibition did not alter the level of p-TBK1 or TBK1 under STING pathway activation.^29^ Instead, PARP7 may affect IFN production through the regulation of NF-κB.^13^ Interestingly, in this study, STAT1/STAT2 protein levels were stabilized in EO771 cells by PARP7 KO or inhibition, which is consistent with our finding.^13^ Our results here showed that PARP7 can directly modify STAT1 and STAT2 and promote their degradation via autophagy, establishing a new mechanism via which PARP7 regulates IFN signaling. Our results, combined with previous reports, suggest that PARP7 not only suppresses the production of IFN by regulating TBK1 or NF-κB but also suppresses downstream IFN signaling by promoting the degradation of STAT1 and STAT2.

PARP7 is known to promote the ubiquitination of several substrate proteins, such as HIF1α, and send them for proteasomal degradation.^20,31,32^ This is achieved through the ADP-ribosylation-dependent phase condensation and recruitment of E3 ligases that can bind to ADP-ribose. We believe that a similar mechanism is operating for PARP7-mediated STAT1 and STAT2 degradation. However, the key difference is that the degradation of STAT1 and STAT2 is through p62-mediated autophagy (Figure 6), while previously reported cases are through proteasomal degradation. Thus, it seems that PARP7, by ADP-ribosylating substrate proteins and recruiting E3 ligases, can promote either proteasomal degradation or autophagy degradation.

IFN therapies are a standard-of-care, first-line treatment for relapsing forms of MS in adults.^33^ Four IFNβ drugs are currently approved to treat relapsing forms of MS.^26^ Given that PARP7 inhibition by RBN can increase IFN-I signaling, as IFNβ would do, we believe that PARP7 inhibitors can play a similar role in MS treatment to IFNβ.^3^ Indeed, RBN treatment alleviates the EAE symptoms in mice and increases STAT1 and p-STAT1 levels in mouse spinal cords. RBN also increases CXCL10 chemokine levels in EAE-induced mice serum compared to DMSO control. Our finding suggests new clinical potential for PARP7 inhibitors, other than the anti-cancer applications previously reported.^13,28,32^ More research and clinical trials should be done to test if PARP7 inhibitors, either alone or in combination with IFNβ, could be used to treat MS.

### Limitations of the study

We have identified STAT1 and STAT2 as substrates of PARP7, although the precise ADP-ribosylation sites on these proteins require further investigation. Additionally, while our research shows that PARP7 recruits p62 and facilitates the degradation of STAT1 and STAT2 via autophagy, the structural mechanisms underlying this process remain unclear. The E3 ligases involved in the ubiquitination of STAT1 and STAT2 also need to be identified. Furthermore, although RBN, a specific PARP7 inhibitor in clinical trials for cancer, has demonstrated efficacy in alleviating MS symptoms in mice, its effectiveness in humans has yet to be confirmed through clinical trials.

## RESOURCE AVAILABILITY

### Lead contact

Further information and requests for resources and reagents should be directed to and will be fulfilled by the lead contact, Hening Lin (linh1@uchicago.edu).

### Materials availability

All unique and stable materials generated in this study are listed in the key resources table and available from the lead contact with a completed materials transfer agreement.

### Data and code availability

- Data reported in this paper will be shared by the lead contact upon request.
- This paper does not report original code.
- Any additional information required to reanalyze the data reported in this paper is available from the lead contact upon request.

## STAR★METHODS

Detailed methods are provided in the online version of this paper and include the following:

### EXPERIMENT MODEL AND STUDY PARTICIPANT DETAILS

#### Mice

C57BL/6 mice were purchased from the Jackson Laboratory (Bar Harbor, ME). Parp7 knock-out mice with C57BL/6 background was generated in this study through editing with CRISPR/Cas9 technology by STEM Cell and Transgenic Mouse Facility of Cornell University. The genes deletion was confirmed with DNA gel electrophoresis and Sanger sequencing. Mouse strains used and generated in this study are also listed in the key resources table. All the mice were kept in a specific-pathogen-free facility and provided with standard laboratory chow, housing in a barrier unit within individually ventilated cages in a room maintained with humidity of 65–75%, 12 h light/dark cycles, and at 22 ± 1°C. 8–10 weeks old mice, matched for age and sex (females only for EAE study), were used for the animal models as described in the method details section below. All the animal experiments were approved by Cornell University’s and University of Chicago’s Institutional Animal Care and Use Committee.

#### Cell culture studies

Cells were either purchased from ATCC or obtained from collaborators without further authentication. All cells were tested mycoplasma negative prior to investigation. HEK293T cells were obtained from the American Type Culture Collection (Manassas, VA). HEK293T were cultured in Dulbecco’s modified Eagle’s medium (Gibco #11965-092) supplemented with 10% heat-inactivated fetal bovine serum (FBS, Gibco #26140079). MEF PARP7^+/+^ PARP^H532A^ and PARP7^−/−^ cells were generated by Jason Matthew’s lab at University of Oslo as previously reported.^28^ MEF cells were cultured in DMEM supplemented with non-essential amino acids (NEAA) and 15% (v/v) heat-inactivated FBS. Cells were grown in a humidified atmosphere at 37°C with 20% O_2_ and 5% CO_2_. Mouse bone marrow-derived macrophages (BMDMs) were differentiated from mouse bone marrow cells, and human peripheral blood mononuclear cells (PBMCs) were ordered from ATCC (PCS-800-011) and differentiated into macrophages following the procedures in the method details section below. All the cells were cultured in a 5% CO_2_ incubator at 37°C.

### METHOD DETAILS

#### Reagents and plasmids

MG132 were purchased from Cayman. CHX and protease inhibitor cocktail tablets EDTA-free were purchased from Sigma-Aldrich. Bafilomycin A1 was purchased from InvivoGen. Pierce Universal Nuclease, Lipofectamine 3000 transfection reagent and Pierce streptavidin magnetic beads were purchased from Thermo Scientific. Lipofectamine RNAiMAX was purchased from Invitrogen. Recombinant mouse IFNβ was purchased from Biotechne R&D Systems. p62 siRNA and negative control siRNA were purchased from Santa Cruz. RBN-2397 was purchased from Ambeed (# A1329221). Poly dA:dT was purchased from Cell Signaling Technology (CST). Hoechst 33342 was purchased from ThermoFisher Scientific (H3570). Pierce protein A/G agarose beads was purchased from from ThermoFisher Scientific (20421).

The plasmids Flag-PARP7, Flag-PARP7 H532A, GFP-PARP7, and GFP-PARP7 H532A used in this paper have been described elsewhere.^20^ pLV-WT-STAT1 was a gift from George Stark (Addgene plasmid # 71454; http://n2t.net/addgene:71454; RRID: Addgene_71454).^24^ pLV-STAT2 was a gift from George Stark (Addgene plasmid # 71451; http://n2t.net/addgene:71451; RRID: Addgene_71451).^25^

#### Cell transfection

For transient overexpression of proteins of interest, HEK293T cells were transfected with expression vectors using PEI MAX 40 K (PolySciences) according to the manufacturer’s protocol. To transfect poly dA:dT, Lipofectamine 3000 transfection reagent (Thermo Scientific) was applied according to the manufacturer’s protocol. To stably knock down p62, 5nM of siRNA was transfected with Lipofectamine RNAiMAX according to the manufacturer’s instructions. Nontarget siRNA was used in control transfections. IFNβ and RBN-2397 were directly added to the cell culture media at indicated concentrations.

#### Western blot

Western blot analysis was performed following previously published methods.^34^ The proteins of interest were detected and visualized using a Typhoon FLA 7000 scanner (GE Healthcare) or ChemiDoc XRS System (Bio-rad). β-actin antibody (C4) (# sc-47778) and GFP Antibody (B-2) (# sc-9996) were purchased from Santa Cruz. STAT1 (9172), phospho-STAT1 (Tyr701) (D4A7) (7649), STAT2 (D9J7L) (72604), phospho-Stat2 (Tyr690) (# 4441), ubiquitin (P4D1) (# 3936), TBK1/NAK (3013), phospho-TBK1/NAK (Ser172) (D52C2) (# 5483), SQSTM1/p62 (# 5114), Poly/Mono-ADP Ribose (D9P7Z) (# 89190), JAK1 (6G4) (# 3344), α-Tubulin (# 2144) antibodies were purchased from Cell Signaling. Monoclonal ANTI-FLAG M2-Peroxidase (HRP) (# A8592) was purchased from MiliporeSigma. Antibodies used in western blotting were diluted 1:1000. PARP7 antibody was obtained from Jason Matthews lab that was previously reported.^28^

#### Quantitative real-time PCR (RT-qPCR)

Total RNA was isolated using E.Z.N.A. Total RNA Kit (VWR) according to the manufacturer’s instructions. Total RNA (0.8 μg) was used for cDNA synthesis using high-capacity cDNA reverse transcription kit (ThermoFisher) following the manufacturer’s instructions. For quantitative real-time PCR analysis, iTaq Universal SYBR Green Supermix (Bio-Rad) was employed following the manufacturer’s instructions. The reaction was conducted using QuantStudioTM 7 Flex Real-Time PCR System (Applied Biosystems). Melting curve analysis was performed to ensure the amplification of a single product in each run. The relative expression of each gene, normalized to GAPDH, was calculated using the 2^−ΔΔCt^ method. Primers used for RT-qPCR are listed in Table S1.

#### Co-IP

To examine the interaction between PARP7 and STAT1/STAT2, HEK 293T cells were seeded in 10-cm plates prior to transfection. On the following day, 5 μg of FLAG-PARP7 WT, H532A, or Flag-empty vector were transfected and cultured overnight. Cells were then collected and lysed in 1% Nonidet P-40 lysis buffer (150 mM NaCl, 25 mM Tris, 1% Nonidet P-40, 10% glycerol, with protease inhibitor and nuclease mixture freshly supplemented). Similar experiment set-up was performed to examine the interaction between PARP7 and p62. To examine the ubiquitination and ADP-ribosylation of STAT1 and STAT2 by PARP7, HEK 293T cells were seeded in 10-cm plates prior to transfection. 5μg of GFP-PARP7 WT, H532A mutant, or GFP-empty vector were co-transfected with Flag-STAT1/STAT2 or Flag-empty vector and cultured overnight. For each sample, 1 mg of whole-cell lysate (quantified with Pierce BCA Protein Assay Kits, purchased from Thermo Scientific) was incubated with 20 μL of anti-FLAG M2 affinity gel for 1.5 h at 4°C under constant mixing. The resulting affinity gel was washed five times with 1 mL of Flag-IP wash buffer (50 mM NaCl, 25 mM Tris, 0.1% Nonidet P-40) and heated in protein loading buffer at 95°C for 10 min. Western blot was performed to detect the interaction of indicated proteins. Flag- and GFP-tagged proteins were detected with HRP-conjugated anti-Flag antibody and anti-GFP antibody (Santa Cruz), respectively. Endogenous STAT1/STAT2 and p62 were detected with STAT1 (# 9172), STAT2 (#D9J7L), and SQSTM1/p62 (# 5114) antibodies purchased from CST, respectively. Ubiquitination was detected by ubiquitin antibody (#P4D1) from CST.

#### Endogenous IP

MEF PARP7 WT and KO Cells were lysed using RIPA lysis buffer (50 mM Tris-HCl, pH 7.4, 150 mM NaCl, 1% NP-40, 0.5% deoxylcholate sodium, 0.1% SDS), and the protein concentration of the resulting lysate was adjusted to 2 mg/mL to obtain a total of 2 mg protein per sample. Lysates were incubated on ice for 30 min with occasional mixing to ensure complete lysis, followed by centrifugation at 13,000 × g for 10 min at 4°C to remove cell debris. To do the pre-clearing, the clarified lysate was incubated with 20 μL of protein A/G agarose beads (Thermo Scientific; # 20421) for 1 h at 4°C with gentle rotation. After incubation, the beads were pelleted by brief centrifugation, and the supernatant was transferred to a new tube for immunoprecipitation. Target-specific antibody (e.g., anti-STAT1/anti-STAT2) or control rabbit IgG (CST; #2729) (was added to the lysate at a volume empirically determined for optimal binding, typically 10 μL antibody per 2 mg of total protein. Samples were incubated overnight at 4°C with gentle rotation. Following antibody incubation, 20 μL of protein A/G agarose beads were added to each sample and incubated for 1 h at 4°C. Immune complexes were collected by centrifugation and washed three times with endogenous-IP wash buffer (50 mM NaCl, 25 mM Tris, 0.1% Nonidet P-40) to reduce nonspecific binding. Bound proteins were eluted by adding 20 μL of 2× SDS loading buffer and heating at 95°C for 5 min. The eluted samples were then subjected to SDS-PAGE and subsequent immunoblot analysis. PARP7 antibody used in endogenous IP is a gift from Jason Matthews, which is previously reported.^28^

#### Streptavidin Pulldown assay

5 × 10^5^ cells of WT or PARP7^−/−^ MEFs were seeded on 6-well plates and incubated in media overnight. The next day cells were lysed with 1% Nonidet P-40 lysis buffer (150 mM NaCl, 25 mM Tris, 1% Nonidet P-40, 10% glycerol, with protease inhibitor and nuclease mixture freshly supplemented). Lysates were collected and incubated in biotin-NAD (Biotechne, R&D Systems # 6573) (6 μg/mL) at 4°C under constant mixing overnight. Cell lysates were then incubated with Pierce streptavidin magnetic beads (Thermo Scientific # 88816) at 4°C under constant mixing for 2 h. The beads were washed five times with 1 mL of Flag-IP wash buffer (50 mM NaCl, 25 mM Tris, 0.1% Nonidet P-40) and heated in protein loading buffer at 95°C for 10 min and analyzed using immunoblot analysis.

#### Immunofluorescence

Cells were seeded in 35-mm glass-bottom dishes (MatTek) and transfected with GFP-PARP7, Flag-STAT1/STAT2, and HA-p62 in HEK 293T cells overnight. Cells were then rinsed with phosphate-buffered saline (PBS) and fixed with 4% paraformaldehyde in PBS for 15 min at room temperature. Fixed cells were washed three times with PBS, permeabilized with 0.1% saponin in PBS, and blocked with 5% bovine serum albumin (BSA) for 30 min at room temperature. Cells were then incubated with indicated antibodies overnight at 4°C in dark in PBS with 0.1% saponin and 5% BSA. Cells were washed with PBS with 0.1% saponin for three times. Samples were then mounted with Fluoromount-G (Southern Biotech #0100-01). Samples were imaged with Zeiss LSM880 inverted confocal microscopy or Zeiss LSM710 confocal microscopy. Images were captured at 63×. Around 100 cells per condition per experiment were analyzed. Experiments were repeated independently three times, and representative images are presented in the figures. Images were processed with Fiji software. The following primary antibodies are used: Alexa Fluor 647 anti-Flag tag antibody at 1:1000 (Invitrogen # MA1-142-A647) and Alexa Fluor 647 anti-HA tag antibody at 1:1000 (Invitrogen # 26183-A647).

#### Expression and Purification of GST-PARP7

pGEX-4T PARP7 was cloned using previously reported Flag-PARP7 plasmid.^20^ Purification of PARP7 followed the protocol previously reported with slight modifications.^35^ Recombinant GST-tagged PARP7 was expressed in *E. coli* strain BL21(DE3) (NEB #C2527H). Bacterial cultures were grown in LB medium containing 100 μg/mL ampicillin at 37°C until reaching an OD_600_ of 0.6–0.8. Protein expression was induced with 1M IPTG for 16 h at 18°C. Cells were harvested by centrifugation (1,000 × g, 10 min, 4°C), resuspended in lysis buffer (20 mM Tris–HCl pH 8.0, 500 mM NaCl, 1 mM EDTA, 1% Triton X-100, 10% glycerol, 2 mM DTT, 1×protease inhibitor cocktail (PIC), 200 μg/mL lysozyme (Thermo Scientific # 89833), 400U DNase I (Stemmcell #07900), and lysed by sonication on ice. Lysates were cleared by centrifugation (20,000 × g, 30 min, 4°C), and the supernatant was passed through Pierce Glutathione Agarose (Thermo Scientific # 16101). The beads were washed extensively with wash buffer (20 mM Tris–HCl pH 8.0, 500 mM NaCl, 1 mM EDTA, 1% Triton X-100, 10% glycerol, 2 mM DTT), and bound proteins were eluted with elution buffer (100 mM Tris–HCl pH 8.0, 150 mM NaCl, 0.2% Tween 20, 10% glycerol, 40 mM GSH). Eluted fractions were analyzed by SDS-PAGE and Coomassie staining to confirm purity. Protein concentration was determined using the BCA assay (Thermo Scientific #23227). Purified GST-PARP7 was aliquoted and stored at −80°C.

#### In vitro ADP-Ribosylation assay

Recombinant human STAT1 (1 μg, MCE #HY-P71335) and STAT2 (1 μg, Sinobiological #S53-54G) were incubated with purified GST-PARP7 (0.5 μg) in ADP-ribosylation reaction buffer containing 50 mM Tris-HCl (pH 8.0), 4 mM MgCl_2_, 0.2 mM DTT, and 100 μM NAD^+^ (Sigma-Aldrich). Reactions were carried out in a total volume of 25 μL at 37°C for 1 h. Reactions were stopped by adding 6x SDS loading dye and heating at 95°C for 5 min. Samples were resolved by SDS-PAGE, transferred to PVDF membranes, and probed with antibodies specific for ADP-ribose (CST # 89190S).

#### Mice

C57BL/6 mice were purchased from the Jackson Laboratories (Bar Harbor, ME). PARP7 knock-out mice with C57BL/6 background were generated in this study through editing with CRISPR/Cas9 technology by STEM Cell and Transgenic Mouse Facility of Cornell University. The genes deletion was confirmed with real-time PCR and genotyping. All the mice were kept in a specific-pathogen-free facility and provided with standard laboratory chow, housing in a barrier unit within individually ventilated cages in a room maintained with humidity of 65–75%, 12 h light/dark cycles, and at 22 ± 1°C. 8–10 weeks old mice, matched for age and sex (usually males and females mixed), were used for the animal models as described in the method details section below. All the animal experiments were approved by Cornell University and University of Chicago’s Institutional Animal Care and Use Committee.

#### Experimental autoimmune encephalomyelitis (EAE)

Female C57BL/6J mice were purchased from the Jackson Laboratory. EAE was induced and scored as previously described.^36^ Briefly, female C57BL/6 mice were immunized subcutaneously with 1 mg/mL myelin oligodendrocyte glycoprotein peptide 35–55 (MOG35-55) (APExBIO #A8306) emulsified at a 1:1 ratio in 1 mg/mL Complete Freud’s Adjuvant, made by mixing killed Mycobacterium tuberculosis H37RA (BD #231141) with incomplete Freund’s adjuvants (Sigma #F5506-6X10ML). On day 0 (starting day) and day 2, 400 ng of pertussis toxin (Enzo # BML-G100-0050) was administered subcutaneously. Starting from day 7, mice were either administered intraperitoneally with DMSO or RBN-2397 (Ambeed # A1329221) at 30 mg/kg every other day. RBN-2397 was completely dissolved in DMSO. Each mouse was injected up to 50uL DMSO or RBN-2397 solution, based on body weight. Clinical scores were evaluated according to the scoring rubric listed previously.^36^ Specifically, the clinical scoring system of EAE mice is shown below. All animal experiments were carried out in accordance with the guidelines of the National Advisory Committee on Laboratory Animal Research and the Cornell University Institutional Animal Care and Use Committee.

**Table:** 

Clinical Scoring System of EAE mice	

Grade	Clinical Design

0	No symptoms
1	Tip of the tail drops
2	Tail (whole) drops
3	Hindlimb paresis and uncoordinated movement
4	One hindlimb paralyzed
5	Two hind limbs paralyzed
6	Two hind limbs paralyzed and uncoordinated forelimbs

#### Cytokine/chemokine measurements

Mice serum from the previous EAE-induced mice were assayed for CXCL10 using Mouse IP-10 (CXCL10) ELISA Kit (Thermo Scientific # BMS6018), IL-10 using Mouse IL-10 ELISA Kit – Quantikine (R&D #M1000B-1), IFNβ using Mouse IFNβ ELISA Kit – Quantikine (R&D # MIFNB0), according to the manufacturer’s instructions.

#### Histological assessment

The spinal cord of mice was quickly dissected after transcranial perfusion (initially with PBS, and then with 4% paraformaldehyde mixed with PBS) at the time of killing. Representative mice were selected from DMSO and RBN-2397 groups or PARP7 WT and KO groups for H&E staining analysis. Tissues were fixed with neutral formalin 10%, embedded in paraffin and serial 8-μm cross-sections were prepared. To investigate immune cell infiltration and myelin loss, three sections of each spinal cord area (cervical, thoracic, and lumbar) were stained with hematoxylin and eosin (H&E) for each animal. The sections at the same position of the spinal cord across different animals were analyzed. The resulting stained tissues were imaged by Olympus VS200 Slideview Research Slide Scanner. Immune cell infiltration was quantified through ImageJ processing software.

#### Immunohistochemistry

Tissues were fixed in 4% paraformaldehyde, embedded in paraffin and cut into 8-μm thick sections for immunohistochemical analysis. After deparaffinization in xylene and rehydration in graded ethanol, the sections were subjected to antigen retrieval. For CD45 (Thermo # 14-0451-82), IBA1 (CST #17198S) and MBP (GeneTex #GTX133108), antigen retrieval was performed by microwave heating in citrate buffer (CST #14746S) for 20 min. Commercially available kit (Abcam #ab64261) and its corresponding protocol were used to complete the following steps. Briefly, after cooling from antigen retrieval, the endogenous peroxidase activity was quenched by 0.3% hydrogen peroxide for 10 min. The tissue section was applied by protein block and incubated with primary antibody diluted in SignalStain Antibody Diluent (CST #8112S) overnight at 4°C with the following dilutions: CD45 at 1:1000; IBA1 at 1:1000; MBP at 1:1500. Negative controls were run in parallel by replacing the primary antibody with rabbit IgG at the equivalent final concentration. On the following day, tissue sections were first incubated in biotinylated goat anti-polyvalent for 10 min, followed by 10 min streptavidin-peroxidase incubation. DAB chromogen and DAB substrate were mixed and added onto tissue sections. Tissues were imaged by Agilent BioTek Cytation 5 cell imaging multi-mode reader. Positive signals were quantified through ImageJ processing software.

### QUANTIFICATION AND STATISTICAL ANALYSIS

Generation of graphs and statistical analysis were done using GraphPad Prism (v10). Statistical evaluation (*p-*values) was done using an unpaired two-tailed Student’s t-test or two-way ANOVA as described in the figure legends. *p* values of <0.05 were considered as statistical significance, indicated by *. Those at *p* ≤ 0.01 are indicated by **, *p* ≤ 0.001 are indicated by ***, and *p* ≤ 0.0001 are indicated by ****. Blots and gel images shown are representative of at least three biological replicates.

## Supplementary Material

1

SUPPLEMENTAL INFORMATION

Supplemental information can be found online at https://doi.org/10.1016/j.celrep.2025.116130.

## Figures and Tables

**KEY RESOURCES TABLE:** 

REAGENT or RESOURCE	SOURCE	IDENTIFIER

Antibodies		

β-actin antibody (C4)	Santa Cruz Biotechnology	Cat# sc-47778; RRID: AB_2714189
GFP Antibody (B-2)	Santa Cruz Biotechnology	Cat# sc-9996; RRID: AB_627695
STAT1	Cell Signaling Technology	Cat# 9172; RRID: AB_2198300
Phosphor-STAT1 (Tyr701) (D4A7)	Cell Signaling Technology	Cat# 7649; RRID: AB_10950970
STAT2 (D9J7L)	Cell Signaling Technology	Cat# 72604; RRID: AB_2799824
Phosphor-STAT2 (Tyr690)	Cell Signaling Technology	Cat# 4441; RRID: AB_2198445
Ubiquitin (P4D1)	Cell Signaling Technology	Cat# 3936; RRID: AB_331292
TBK1/NAK	Cell Signaling Technology	Cat# 3013; RRID: AB_2199749
phospho-TBK1/NAK (Ser172) (D52C2)	Cell Signaling Technology	Cat# 5483; RRID: AB_10693472
SQSTM1/p62	Cell Signaling Technology	Cat# 5114; RRID: AB_10624872
Poly/Mono-ADP Ribose (D9P7Z)	Cell Signaling Technology	Cat# 89190; RRID: N/A
JAK1 (6G4)	Cell Signaling Technology	Cat# 3344; RRID: AB_2265054
α-Tubulin	Cell Signaling Technology	Cat# 2144; RRID: AB_2210548
Control rabbit IgG	Cell Signaling Technology	Cat# 2729; RRID: AB_1031062
IBA1	Cell Signaling Technology	Cat# 17198; RRID: AB_2820254
Monoclonal ANTI-FLAG^®^ M2-Peroxidase (HRP)	MiliporeSigma	Cat# A8592; RRID: AB_439702
Alexa Fluor 647 anti-Flag tag antibody	Invitrogen	Cat# MA1-142-A647; RRID: AB_2610655
Alexa Fluor 647 anti-HA tag antibody	Invitrogen	Cat# 26183-A647; RRID: AB_2610626
CD45	Thermofisher Scientific	Cat# 14-0451-82; RRID: AB_467251
MBP	GeneTex	Cat# GTX133108; RRID: AB_2886829
PARP7	This study (Rusmussen et al.)^28^	N/A

Biological samples		

Mouse serum	C57BL/6-PARP7^+/+^ and PARP7^−/−^ mice	N/A
Mouse spinal cords	C57BL/6-PARP7^+/+^ and PARP7^−/−^ mice	N/A

Chemicals, peptides, and recombinant proteins		

Mg132	Cayman Chemical	Cat# 10012628; CAS# 133407-82-6
Cycloheximide (CHX)	Sigma-Aldrich	Cat# C7698; CAS# 66-81-9
Incomplete Freund’s adjuvants	Sigma-Aldrich	Cat# #F5506-6X10ML
NAD+	Sigma-Aldrich	Cat# TA9H9A9A4512; CAS: 53-84-9
Bafilomycin A1	InvivoGen	Cat# tlrl-baf1; CAS: 88899-55-2
poly (dA:dT)	InvivoGen	Cat# tlrl-patn; CAS: 86828-69-5
SQSTM1/p62 siRNA (m)	Santa Cruz Biotechnology	Cat# sc-29828
Control siRNA-A	Santa Cruz Biotechnology	Cat# sc-37007
DMEM, high glucose	Thermofisher Scientific	Cat# 11965126
Fetal Bovine Serum, certified, United States	Thermofisher Scientific	Cat# 16000069
Antibiotic-Antimycotic (100X)	Thermofisher Scientific	Cat# 15240062
GlutaMAX^™^ Supplement	Thermofisher Scientific	Cat# 35050061
Opti-MEM^™^ | Reduced Serum Medium	Thermofisher Scientific	Cat# 31985070
MEM Non-Essential Amino Acids Solution (100X)	Thermofisher Scientific	Cat# 11140050
Lysozyme	Thermofisher Scientific	Cat# 89833; CAS# 9001-63-2
Recombinant Mouse M-CSF (carrier-free)	Biolegend	Cat# 576406
Polyethylenimine Hydrochloride (PEI)	Polysciences	Cat# 24885-2
RBN-2397	Ambeed	Cat# A1329221; CAS# 2381037-82-5
Recombinant human STAT1	MedChemExpress	Cat# HY-P71335
Recombinant human STAT2	SinoBiological	Cat# S53-54G
Recombinant Mouse IFN-beta Protein	Biotechne	Cat# 8234-MB
DNase I Solution	Stemmcell Technologies	Cat# 07900
MOG (35-55)	APExBIO	Cat# A8306; CAS# 149635-73-4
Pertussis toxin (Bordetella pertussis)	Enzo Life Sciences	Cat# # BML-G100-0050; CAS# 70323-44-3
Mycobacterium tuberculosis H37RA	Becton Dickinson	Cat# 231141

Critical commercial assays		

Mouse IP-10 (CXCL10) ELISA Kit	Thermofisher Scientific	Cat# BMS6018
Lipofectamine^™^ RNAiMAX Transfection Reagent	Thermofisher Scientific	Cat# 13778075
Rabbit specific HRP/DAB Detection IHC Kit	Abcam	Cat# ab64261
Mouse IL-10 ELISA Kit – Quantikine	R&D Systems	Cat# M1000B-1
Mouse IFNβ ELISA Kit – Quantikine	R&D Systems	Cat# MIFNB0
E.Z.N.A.^®^ Total RNA Kit I	Omega BIO-TEK	Cat# R6834-02
Applied Biosystems^™^ High-Capacity cDNA Reverse Transcription Kit	Fisher Scientific	Cat# 43-688-13
2X Universal SYBR Green Fast qPCR Mix	Abclonal	Cat# RK21203
ANTI-FLAG^®^ M2 Affinity Gel	Sigma-Aldrich	Cat# A2220
Protein A/G conjugated agarose beads	Santa Cruz Biotechnology	Cat# sc-2003

Experimental models: Cell lines		

HEK293T	ATCC	ATCC# CRL-11268
MEF PARP7-WT	This study (Rusmussen et al.)^28^	N/A
MEF PARP7-KO	This study (Rusmussen et al.)^28^	N/A
MEF PARP7-H532A	This study (Rusmussen et al.)^28^	N/A

Experimental models: Organisms/strains		

Mouse: C57BL/6 PARP7^−/−^	STEM Cell and Transgenic Mouse Facility of Cornell University, this study.	N/A

Oligonucleotides		

qPCR primers	This paper	Table S1

Recombinant DNA		

Flag-empty vector	This paper (Zhang et al.)^20^	N/A
Flag-PARP7 (WT)	This paper (Zhang et al.)^20^	N/A
Flag-PARP7 (H532A)	This paper (Zhang et al.)^20^	N/A
GFP-PARP7 (WT)	This paper (Zhang et al.)^20^	N/A
GFP-PARP7 (H532A)	This paper (Zhang et al.)^20^	N/A
GFP-empty vector	This paper (Zhang et al.)^20^	N/A
pLV-WT-STAT1	Addgene	Cat# 71454; RRID: Addgene_71454
pLV-STAT2	Addgene	Cat# 71451; RRID: Addgene_71451

Software and algorithms		

Image Lab 6.1	Bio-Rad Laboratories, Inc.	https://www.bio-rad.com/en-us/product/image-lab-software?ID=KRE6P5E8Z#fragment-3
GraphPad Prism Version 9.5.0 (730)	GraphPad software, inc.	https://www.graphpad.com/
BioRender	BioRender	https://www.biorender.com
ImageJ software	Fiji	https://imagej.net/software/fiji/downloads
Zotero 6.0.23	Zotero, Corporation for Digital Scholarship.	https://www.zotero.org/
ZEN (blue edition)	Carl Zeiss Microscopy GmbH	https://www.zeiss.com/corporate/us/home.html

## Highlights

- PARP7 regulates the type I interferon signaling pathway
- PARP7 forms cytosolic foci and ADP-ribosylates STAT1/STAT2
- PARP7 promotes STAT1/2 ubiquitination and p62-mediated autophagic degradation
- Inhibition of PARP7 relieves experimental autoimmune encephalomyelitis in mice
