# Supplementary material for: PARP7 inhibition stabilizes STAT1/STAT2 and relieves experimental autoimmune encephalomyelitis in mice
Source: Cell Rep. Author manuscript; Available in PMC 2025 Oct 9. (PMC12509220; doi:10.1016/j.celrep.2025.116130)
Supplement: 1 [file NIHMS2107283-supplement-1.pdf]

Cell Reports, Volume *44*

Supplemental information

PARP7 inhibition stabilizes STAT1/STAT2 and relieves experimental autoimmune encephalomyelitis in mice

Jiashu Xu, Tao Yu, Zongwei Yue, Xuan Lu, Yandong Zhang, Lei Wang, Samaneh Shabani Åhrling, Michael R. Smith, Yan Chun Li, Jason Matthews, and Hening Lin

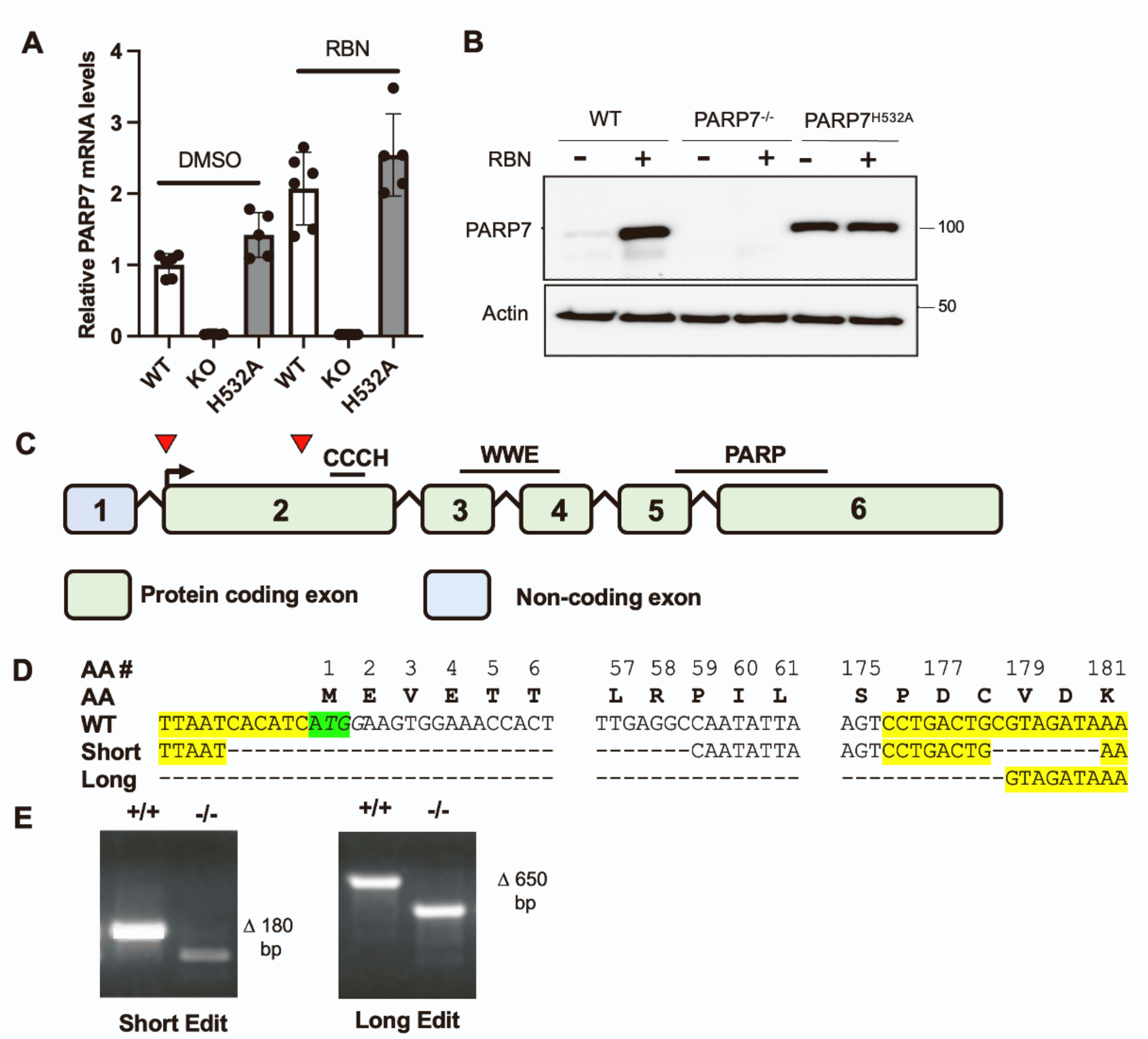

Figure S1. Characterization of *Parp7*$^{-/-}$/ *Parp7*^{H532A} MEF cells and *Parp7*$^{-/-}$ mice. (A) Quantification of *Parp7* mRNA levels by RT-qPCR in wild-type (WT), *Parp7* knockout (*PARP7*$^{-/-}$), and catalytically inactive *Parp7 H532A* (PARP^{H532A}) MEF cells treated with either DMSO or 100 nM RBN for 24h. *Parp7* mRNA levels are normalized to a housekeeping gene and expressed relative to WT DMSO control. Each dot represents an independent biological replicate ($n \geq 4$); bars show mean ± SEM. (B) Immunoblot analysis of PARP7 protein expression in WT, *Parp7*$^{-/-}$, and *Parp7*^{H532A} cells treated with 100 nM RBN for 24h. Actin serves as a loading control. Arrow indicates PARP7 protein band. (C) Schematic of gRNAs for *Parp7* gene targeting exon 2. (D) Sequencing results of *Parp7* genotyping from mice with either long or short edit. (E) DNA gel to visualize either short or long edit compared to WT mice.

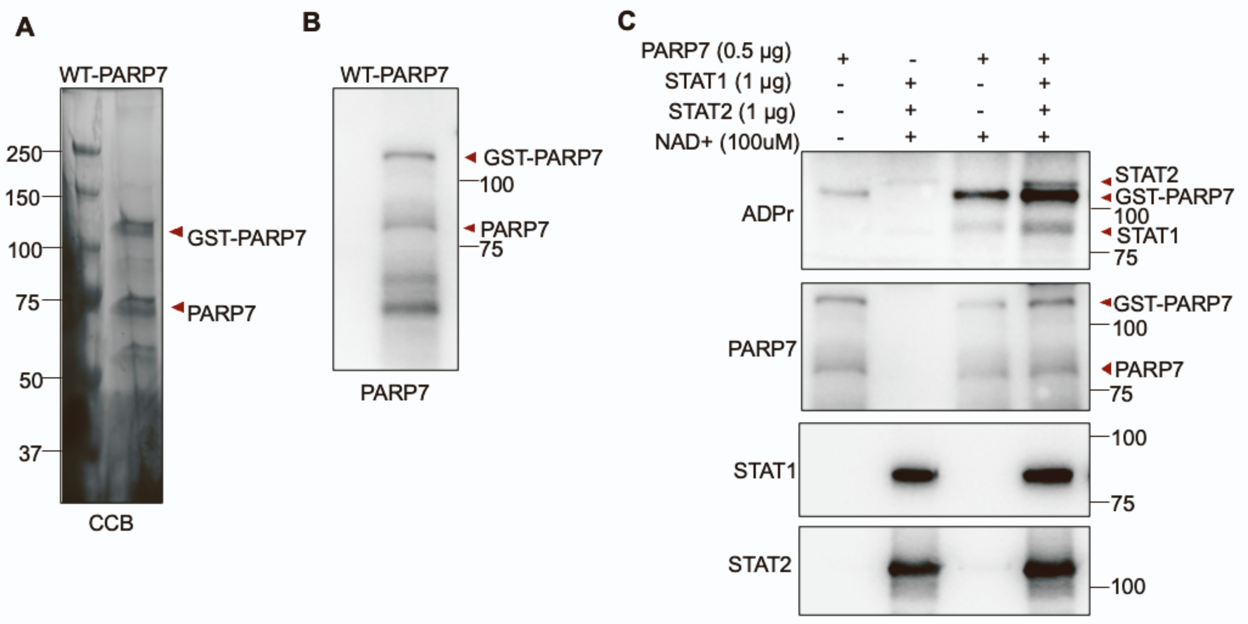

Figure S2: Characterization of PARP7 mono-ADP-ribosyltransferase activity on STAT1/STAT2. (A) Coomassie Brilliant Blue (CCB) stain of recombinant WT PARP7 protein showing both full-length GST-tagged and cleaved PARP7 bands after purification. **(B)** Immunoblot detection of wild-type PARP7 using an anti-PARP7 antibody confirming the presence of both GST-tagged and cleaved forms of PARP7.**(C)** In vitro ADP-ribosylation assay assessing the modification of STAT1 and STAT2 by PARP7. Reactions were performed with or without recombinant GST-PARP7 (0.5 µg), STAT1 (1 µg), STAT2 (1 µg), and NAD^+ (100 µM) as indicated. ADP-ribosylation (ADPr) was detected by immunoblotting using a pan-ADP-ribose antibody. SE: short exposure; LE: long exposure. Expression of GST-PARP7, STAT1, and STAT2 was confirmed by immunoblotting. Experiment was repeated three times.

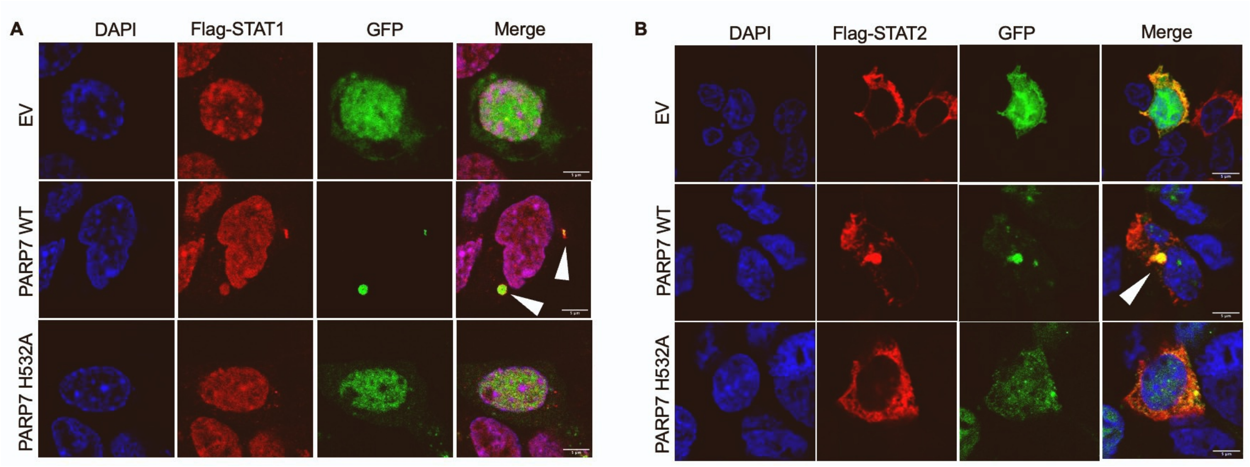

Figure S3. PARP7 forms nuclear foci and recruits STAT1/STAT2. (**A**) HEK 293T cells were co-transfected with Flag-STAT1 as well as empty vector (GFP), GFP-tagged WT, or H532A mutant PARP7 overnight with 5 μM Bafilomycin A1 treatment. Flag-STAT1 was detected by immunofluorescence with anti-FLAG (red) antibodies. Nuclei were stained with Hoeschst stain (blue) (scale bar, 5 μm.) (**B**) HEK 293T cells were co-transfected with Flag-STAT2 as well as empty vector (GFP), GFP-tagged WT, or H532A mutant PARP7 with 5 μM Bafilomycin A1 treatment. Flag-STAT2 was detected by immunofluorescence with anti-FLAG (red) antibodies. Nuclei were stained with Hoeschst stain (scale bar, 5 μm.)

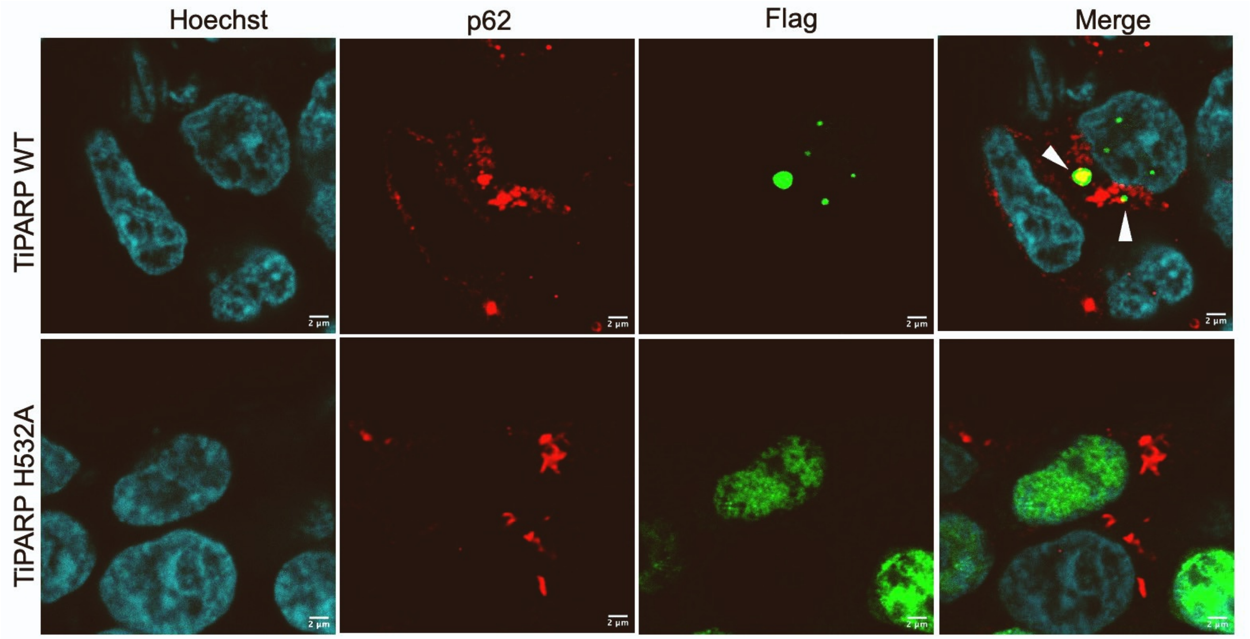

Figure S4. PARP7 enzymatic activity promotes co-localization with p62 in cells. HEK293T cells were transiently transfected with either Flag-tagged wild-type PARP7 (PARP7 WT) or catalytic mutant PARP7 (H532A). Cells were fixed, permeabilized, and stained for Flag (green), p62 (red), and DNA (Hoechst, blue). Confocal microscopy images show that Flag-PARP7 WT forms distinct puncta that strongly co-localize with p62 (white arrowheads in Merge panel), whereas PARP7 H532A displays diffuse nuclear and cytoplasmic localization with minimal co-localization with p62. Scale bar, 2 μm.

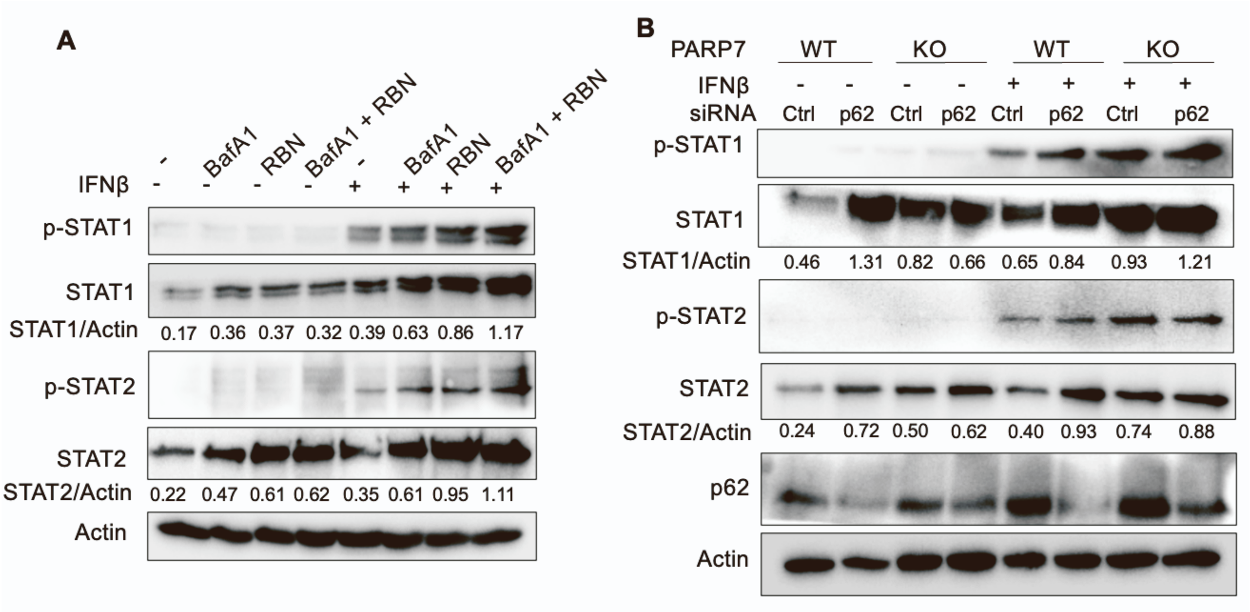

Figure S5. PARP7 regulates IFNβ-induced STAT1/2 signaling through autophagy pathways. (A) Immunoblot analysis of phosphorylated STAT1 (p-STAT1), endogenous STAT1, phosphorylated STAT2 (p-STAT2), and endogenous STAT2 in cells treated with IFNβ in the presence or absence of autophagy inhibitors Bafilomycin A1 (BafA1, 5uM) and/or RBN-2397 (RBN, 100 nM). Quantifications of STAT1/Actin, and STAT2/Actin ratios are shown below each blot to indicate relative expression levels normalized to loading controls. **(B)** Immunoblot analysis of p-STAT1, STAT1, p-STAT2, STAT2, and p62 in wild-type (WT) or PARP7 knockout (KO) cells with or without IFNβ stimulation and transfected with control siRNA (siCtrl) or p62 siRNA (sip62). Actin is shown as a loading control. Quantifications of STAT1/Actin, and STAT2/Actin ratios are shown below each blot to indicate relative expression levels normalized to loading controls.

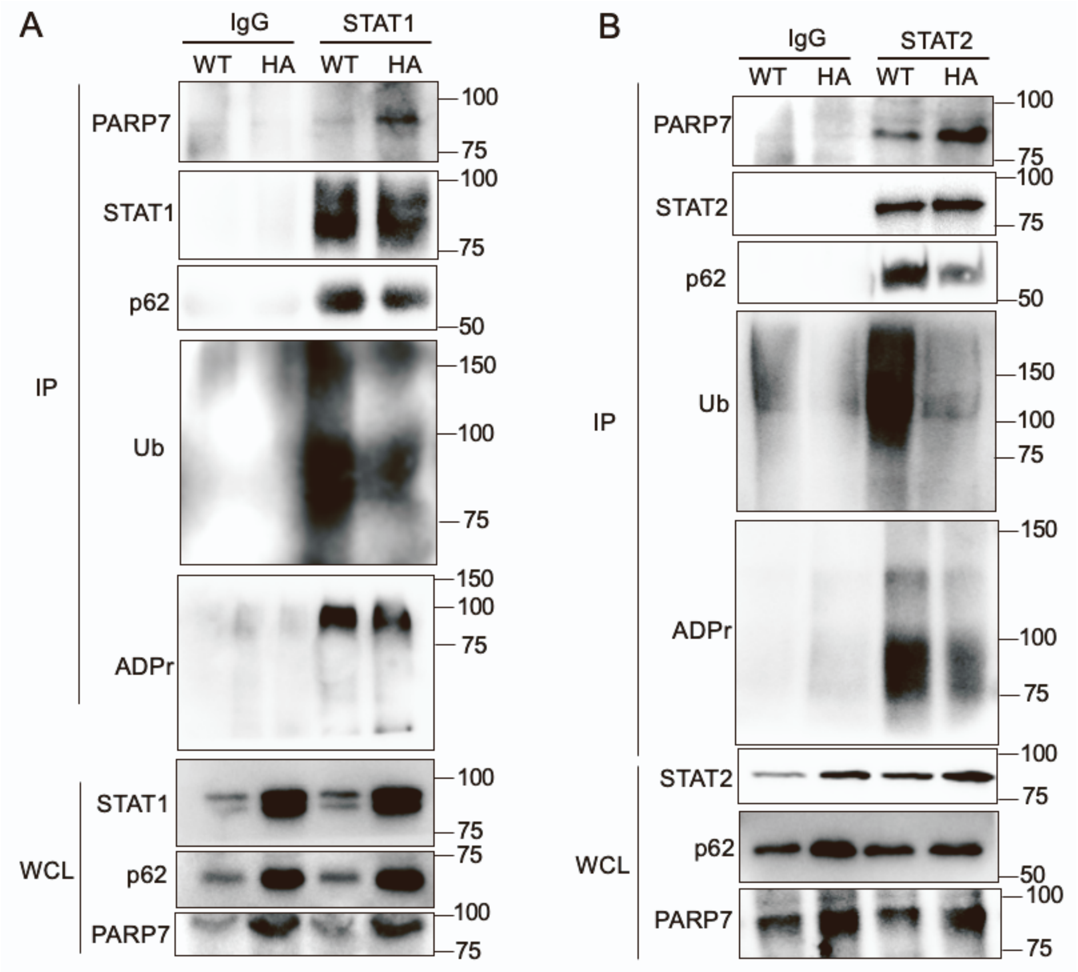

Figure S6. PARP7 interacts with STAT1 and STAT2 and promotes their ubiquitination and ADP-ribosylation. (A, B) Co-immunoprecipitation (IP) and immunoblotting (IB) analysis of protein interactions and post-translational modifications in wild-type (WT) and PARP7-overexpressing (HA) cells. **(A)** STAT1 was immunoprecipitated from WT and H532A MEF cells, with IgG used as a control. Immunoblots were probed for PARP7, STAT1, p62 (autophagy adaptor), ubiquitin (Ub), and ADP-ribose (ADPr). Whole-cell lysates (WCL) were also analyzed to assess total protein expression levels. **(B)** STAT2 was immunoprecipitated from WT and H532A MEF cells, with IgG used as a control. Immunoblots were probed for PARP7, STAT2, p62 (autophagy adaptor), ubiquitin (Ub), and ADP-ribose (ADPr). Whole-cell lysates (WCL) were also analyzed to assess total protein expression levels.

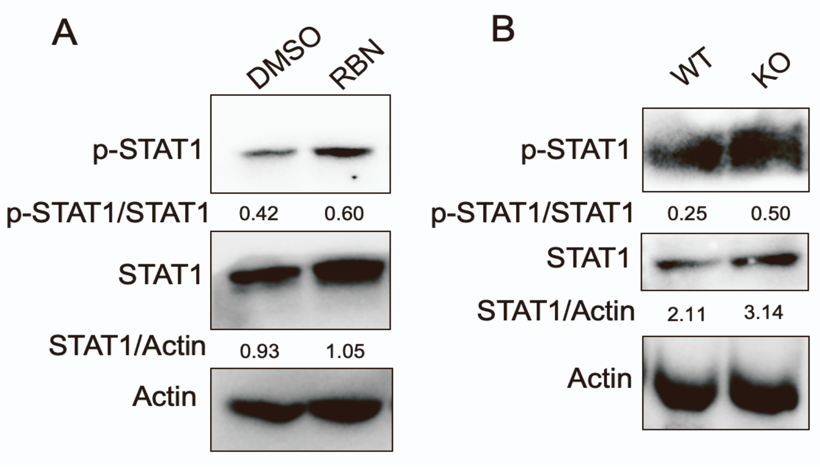

Figure S7. STAT1 and phosphorylated STAT1 (p-STAT1) increase in spinal cord lysates of RBN treatment and PARP7 deletion in C57BL/6 mice. (A) Western blot analysis of spinal cord lysates from DMSO- and RBN-treated mice. RBN treatment increases the p-STAT1/STAT1 ratio compared to DMSO (0.60 vs. 0.42), indicating enhanced STAT1 phosphorylation. Total STAT1 also increases in RBN-treated mice (1.05 vs 0l93). β-Actin were used as loading controls, and STAT1/Actin ratios are shown below each lane. **(B)** Western blot of spinal cord lysates from wild-type (WT) and knockout (KO) mice. KO mice exhibit an increased p-STAT1/STAT1 ratio (0.50 vs. 0.25), and elevated total STAT1 levels (STAT1/Actin ratio: 3.14 vs. 2.11) compared to WT. β-Actin serves as a loading control.

Table S1. Primers for RT-qPCR

Target gene	Sequence
Mouse *CXCL10*	ATCATCCCTGCGAGCCTATCCT
	GACCTTTTTTGGCTAAACGCTTTC
Mouse *GAPDH*	CATCACTGCCACCCAGAAGACTG
	ATGCCAGTGAGCTTCCCGTTCAG
Mouse *ISG15*	CATCCTGGTGAGGAACGAAAGG
	CTCAGCCAGAACTGGTCTTCGT
